# Evolution, Development and Function of Vertebrate Cone Oil Droplets

**DOI:** 10.3389/fncir.2017.00097

**Published:** 2017-12-08

**Authors:** Matthew B. Toomey, Joseph C. Corbo

**Affiliations:** Department of Pathology and Immunology, Washington University School of Medicine, St. Louis, MO, United States

**Keywords:** color vision, spectral sensitivity, carotenoids, visual ecology, photoprotection, cone photoreceptor, dim-light vision

## Abstract

To distinguish colors, the nervous system must compare the activity of distinct subtypes of photoreceptors that are maximally sensitive to different portions of the light spectrum. In vertebrates, a variety of adaptations have arisen to refine the spectral sensitivity of cone photoreceptors and improve color vision. In this review article, we focus on one such adaptation, the oil droplet, a unique optical organelle found within the inner segment of cone photoreceptors of a diverse array of vertebrate species, from fish to mammals. These droplets, which consist of neutral lipids and carotenoid pigments, are interposed in the path of light through the photoreceptor and modify the intensity and spectrum of light reaching the photosensitive outer segment. In the course of evolution, the optical function of oil droplets has been fine-tuned through changes in carotenoid content. Species active in dim light reduce or eliminate carotenoids to enhance sensitivity, whereas species active in bright light precisely modulate carotenoid double bond conjugation and concentration among cone subtypes to optimize color discrimination and color constancy. Cone oil droplets have sparked the curiosity of vision scientists for more than a century. Accordingly, we begin by briefly reviewing the history of research on oil droplets. We then discuss what is known about the developmental origins of oil droplets. Next, we describe recent advances in understanding the function of oil droplets based on biochemical and optical analyses. Finally, we survey the occurrence and properties of oil droplets across the diversity of vertebrate species and discuss what these patterns indicate about the evolutionary history and function of this intriguing organelle.

## Introduction

“When you observe a fragment of [avian] retina from the outside, you see one of the most beautiful sights the microscope can afford: the entire field of view is bedecked with tiny globules of different colors.” [authors’ translation from the German] (Hannover, 1840).

**Figure 1:**
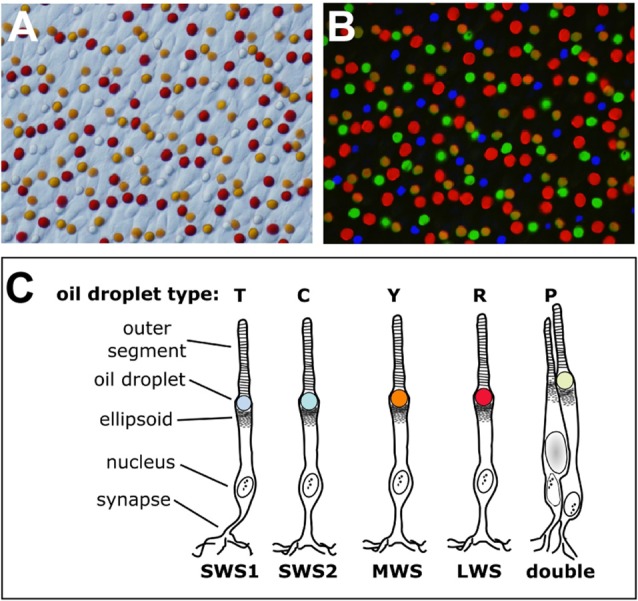
The cone oil droplets of the vertebrate retina. **(A)** The cone oil droplets of the painted turtle (*Chrysemys picta*) are shown here in a bright field image of a flat-mounted retina, 400× magnification. **(B)** Overlaid in this image is the same retinal field as in **(A)** viewed with ultraviolet (327 nm), blue (460–490 nm) and green (520–550 nm) epifluorescent illumination. Note that droplets within each cone photoreceptor subtype have distinct patterns of fluorescent excitation and emission that permit five different subtypes to be distinguished. The T-type droplets do not fluoresce because they lack carotenoid pigmentation. **(C)** A schematic representation of the cone photoreceptors of the chicken (*Gallus gallus*). The cone subtypes are identified by their visual pigment opsins. The nomenclature commonly used for the different oil droplet types is also indicated. This drawing is adapted from Toomey et al. (2015).

Cone oil droplets have been a subject of enduring aesthetic fascination and scientific curiosity for nearly 200 years, as indicated by the observations of Adolph Hannover (above) published in 1840 (Hannover, 1840; Goldsmith et al., 1984). oil droplets are spherical optical organelles (between 1.6 μm and 14 μm in diameter, depending on the cone subtype and species) that reside within the sclerad portion of the cone photoreceptor inner segment of a wide range of vertebrate species (Figure 1; Ives et al., 1983; Goldsmith et al., 1984). The droplets consist of colorless neutral lipids pigmented with a range of carotenoids that endow the droplets of different cone subtypes with colors ranging from transparent to brilliant red (Goldsmith et al., 1984). Recent studies indicate that oil droplets have two main functions: they act as intracellular microlenses that enhance light delivery to the outer segment (Stavenga and Wilts, 2014; Wilby et al., 2015; Wilby and Roberts, 2017); and they filter the spectrum of light reaching the outer segment, thereby improving color discrimination and color constancy (Vorobyev et al., 1998; Vorobyev, 2003; Olsson et al., 2016). Enhancement of light delivery is primarily observed in colorless droplets, since light-filtering by carotenoids reduces the amount of light reaching the outer segment, thereby negating the lensing effect. While the stunningly pigmented oil droplets of birds and turtles have garnered the most scientific attention, these organelles are widely, yet patchily, distributed among vertebrates, occurring in five of the seven extant vertebrate classes. Cone oil droplets are notably absent from the retinas of placental mammals, including humans, which is one reason they have escaped the attention of many modern vision scientists. In this review, we will briefly consider the colorful history of oil droplet research, speculate about the developmental origins of droplets, discuss recent advances in understanding their composition and function, and present a survey of the distribution and properties of oil droplets among diverse vertebrate clades (throughout this review, “clade” is meant to indicate a group of organisms that have evolved from a common ancestor).

In the early years of oil droplet research, numerous aspects of their biology were studied, including color (Waelchli, 1883), pigment content (Wald and Zussman, 1938), spatial and species distribution (Peiponen, 1964; Muntz, 1972) and function (Walls and Judd, 1933; Muntz, 1972). Early theories of oil droplet function proposed that droplets might: enhance visual acuity by reducing chromatic aberration; improve visual contrast; reduce glare; or protect photoreceptors against damage by short-wavelength light (Muntz, 1972). One theory that was particularly favored suggested that vertebrates possess only a single visual pigment and that oil droplets of varying color act as spectral pre-filters that deliver a modified spectrum of light to the pigment, thereby permitting wavelength discrimination (Roaf, 1933; Wald, 1938; Donner, 1960; Hailman, 1964). This theory was put to rest once the presence of multiple cone pigments was demonstrated in birds and turtles (Liebman and Granda, 1971; Bowmaker and Knowles, 1977).

Early oil droplet studies were hampered by subjective descriptions of oil droplet colors, which were difficult to reproduce across microscopes and observers. Nonetheless, chemical analyses of whole-retina extracts showed that oil droplets contained carotenoids (Wald and Zussman, 1938), and early microspectrophotometric (MSP) studies revealed that differently colored droplets absorbed nearly all light below a defined “cut-off” wavelength, transmitting all longer wavelengths (Roaf, 1929; Strother, 1963; Muntz, 1972). Despite such progress, the high carotenoid concentration of individual droplets precluded the measurement of detailed absorbance spectra that would allow unequivocal determination of the chemical identity of the carotenoids within individual droplet types. This problem was eventually solved by Liebman and Granda (1975), who used MSP to measure the absorption of individual turtle oil droplets that had been fused with larger droplets of mineral oil and thereby sufficiently diluted to permit accurate measurement of carotenoid spectra. This technique was subsequently utilized by Goldsmith et al. (1984) to define the carotenoid content of multiple oil droplet types from 19 species of bird. These groundbreaking studies paved the way for our current understanding of oil droplet structure and function.

## Development of Oil Droplets

Little is known about the development of cone oil droplets. During chicken (*Gallus gallus*) embryogenesis, oil droplets first appear above the optic nerve head around embryonic day 10 (E10) as minute colorless globules (López et al., 2005). Droplet differentiation and growth proceed outward from there, following the pattern of photoreceptor neurogenesis. Droplet pigmentation does not begin until E16–17, and full growth and differentiation is not complete until around post-hatch day 15. Gene expression profiling of developing chicken cone subtypes revealed a number of genes with possible roles in oil droplet formation (Enright et al., 2015). However, no detailed mechanistic studies of droplet development have been performed. Thus, the molecular underpinnings of oil droplet formation remain unknown.

The organellar origin of oil droplets is similarly obscure. In most species with oil droplets, the distal cone inner segment immediately adjacent to the droplet contains an aggregate of tightly packed mitochondria referred to as the “ellipsoid” (MacNichol et al., 1978; Nag and Bhattacharjee, 1995). The proximity of the oil droplet to these mitochondrial aggregates suggests that oil droplets might be highly modified mitochondria. However, there is no molecular or ultrastructural evidence to support this notion. In some species, particularly those without oil droplets, the ellipsoidal mitochondria can be massively enlarged (Nag and Bhattacharjee, 1995; Knabe et al., 1997; Lluch et al., 2003; Tarboush et al., 2014). These so-called “megamitochondria” have reduced numbers of cristae and a highly dense appearance in electron micrographs. A recent study in zebrafish implicated a phylogenetically conserved protein, ES1, in the process of megamitochondrion formation (Masuda et al., 2016), but little else is known about their development at the molecular level. In some species of freshwater fish, megamitochondria assume a spherical, highly electron-dense configuration, and are referred to as “ellipsosomes” (MacNichol et al., 1978; Nag and Bhattacharjee, 1995). These structures retain histochemical features characteristic of mitochondria and do not stain with oil-soluble dyes (MacNichol et al., 1978). Ellipsosomes may play light-collecting and spectral filtering roles similar to oil droplets (see below) and thus may represent an instance of convergent evolution, but they appear to be developmentally distinct.

An alternative hypothesis as to the organellar origin of oil droplets is that they derive from lipid droplets, minute cellular organelles found in many cell types and involved in the regulation of intracellular lipid storage and metabolism (Hashemi and Goodman, 2015; Welte, 2015; Onal et al., 2017). The pigeon (*Columba livia*) retina provides indirect support for this hypothesis. In the so-called “red area” of the pigeon retina, the red-sensitive single cones, in addition to a large red oil droplet, contain numerous minute orange and red-pigmented “microdroplets” in the inner segment (Walls and Judd, 1933; Pedler and Boyle, 1969; Mariani and Leure-duPree, 1978), which are comparable in size to lipid droplets. This unusual arrangement might represent an arrested, neotenic state of oil droplet development from lipid droplets. This hypothesis could be tested by examining mature oil droplets for the presence of molecular markers of lipid droplets and via detailed analysis of oil droplet development during embryogenesis.

## Cone Oil Droplet Composition and Carotenoid Metabolism

Our current understanding of the non-carotenoid components of cone oil droplets is limited. Johnston and Hudson (1976) studied the chicken in the only detailed analysis of neutral lipid composition of oil droplets to date. They found that the oil droplets are composed of cholesterols and mono-, di- and triacylglycerols specifically enriched for polyunsaturated fatty acids including linoleic and arachidonic acid (Johnston and Hudson, 1976). Phospholipids were absent from their oil droplet preparations, suggesting that the oil droplets may lack phospholipid membranes (Johnston and Hudson, 1974, 1976). However, an early electron microscopic study indicated that oil droplets are membrane-bound (Morris and Shorey, 1967). An in-depth ultrastructural analysis, including determination of whether oil droplets are surrounded by a phospholipid monolayer (like lipid droplets) or a bilayer (like mitochondria), has not been performed. Clearly, additional investigations are needed to elucidate the lipid composition and ultrastructure of oil droplets.

The cone oil droplets of many species are pigmented with carotenoids, and droplets have correspondingly emerged as a model system for understanding the mechanisms of carotenoid metabolism and transport in vertebrates (Toomey et al., 2015, 2016; Toews et al., 2017). Carotenoids are isoprenoid molecules consisting of an extended system of conjugated double bonds. Electron delocalization along this conjugated system permits absorption of light in the visible range (Goodwin, 1984). The absorption of specific wavelengths is determined, in part, by the number of double bonds in the system (Goodwin, 1984; Chábera et al., 2009). Carotenoids can reach extremely high densities within cone oil droplets. For example, the optical density of red droplets in some turtle species has been reported to be as high as 90 units at the absorbance peak (Liebman and Granda, 1975), meaning that these droplets absorb essentially all light below their cutoff wavelength and therefore act as excellent long-pass filters. Typically carotenoid concentration in individual oil droplets varies according to subtype, with the highest concentrations observed in red droplets and progressively lower concentrations in Y-type, C-type and P-type droplets (Goldsmith et al., 1984; Toomey et al., 2015).

**Figure 2:**
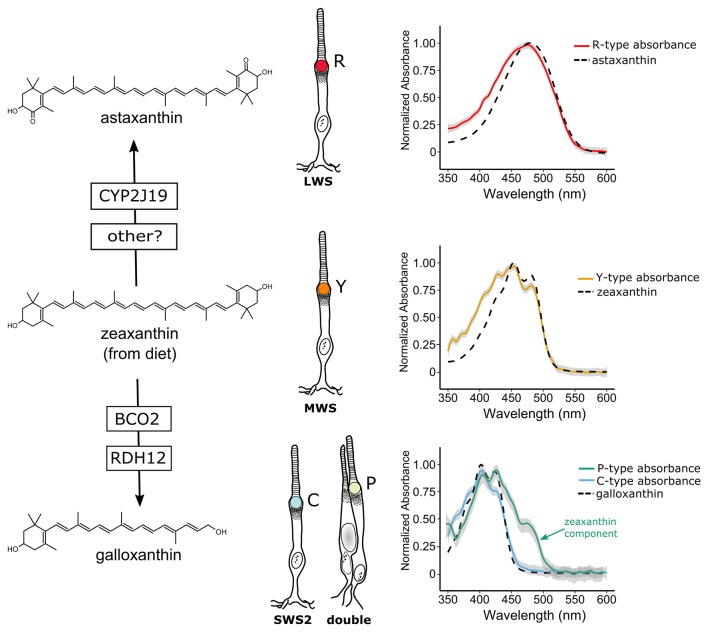
Avian cone oil droplets are pigmented with carotenoids that are selectively metabolized in various cone photoreceptor subtypes. On the left, the molecular structure of the primary carotenoid pigments in the R-type (astaxanthin), Y-type (zeaxanthin) and the C and P-type droplets (galloxanthin) are shown. Astaxanthin is formed through the 4,4′ ketolation of diet-derived zeaxanthin, a transformation that is thought to be mediated, at least in part, by the cytochrome P450 enzyme, CYP2J19 (Lopes et al., 2016). The apocarotenoid galloxanthin is formed through the oxidative cleavage of zeaxanthin and subsequent reduction of the resulting aldehyde. The enzymes β-carotene oxygenase 2 (BCO2) and retinol dehydrogenase 12 (RDH12) have been implicated in the formation of galloxanthin in the avian retina (Toomey et al., 2016). On the right, the light absorbance spectra of pure astaxanthin, zeaxanthin and galloxanthin are overlaid on the absorbance spectra of expanded oil droplets from each of the pigmented cone oil droplets of the chicken (shown as mean ± SD of individual normalized spectra, *n* = 4–5). The spectra of the oil droplets deviate somewhat from the spectra of the dominant carotenoid due to the presence of small quantities of other carotenoid types in each droplet (Toomey et al., 2015). For example, the long-wavelength shoulder seen in the P-type droplet spectrum is attributable to the presence of small amounts of lutein and zeaxanthin in addition to galloxanthin. Portions of this figure are adapted from Toomey et al. (2015).

**Figure 3:**
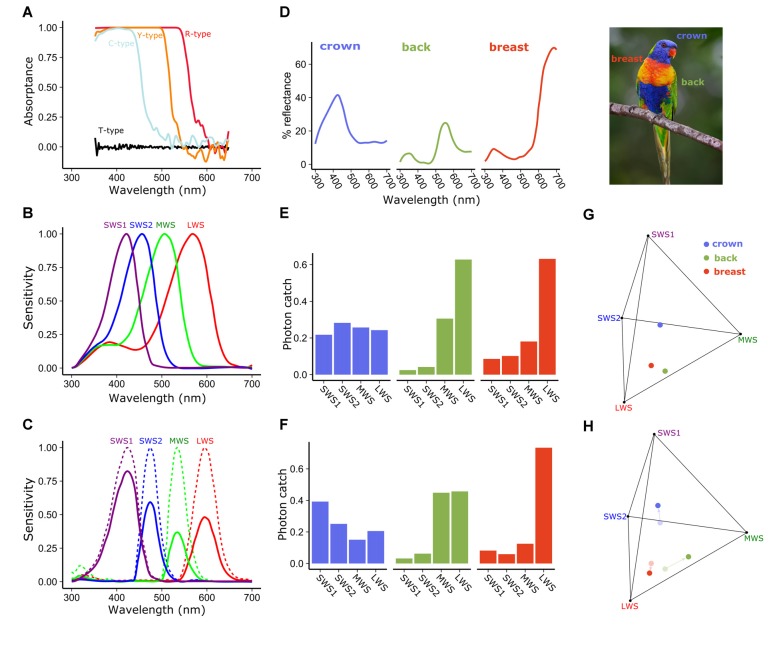
Spectral filtering by cone oil droplets modifies receptor sensitivity and facilitates color discrimination. **(A)** The cone oil droplets of three of the four single cones of the chicken retina function as long-pass cut-off filters, absorbing nearly all light below the cut-off wavelength. These absorptance spectra are calculated based on measures of expanded oil droplets and estimated optical density of the unexpanded oil droplets. Note that the fine structure of the carotenoid spectra seen in Figure 2 are obscured by the high pigment concentrations in the unexpanded droplet spectra shown here. **(B)** The normalized spectral sensitivities of the opsins of the four single cones of the chicken retina without cone oil droplet filtering are relatively broad and overlapping. **(C)** Subtracting light absorbance and scattering by the oil droplets (as shown in **A**) from the opsin sensitivities (shown in **B**) results in substantially narrowed and reduced spectral sensitivities of cone photoreceptors. Solid lines represent the sensitivity spectra of the droplet-filtered cones, and the dotted lines are these same spectra normalized to their peak sensitivity. To illustrate the influence of this spectral filtering on color discrimination we calculated the photon catch of photoreceptors viewing the colored plumage patches **(D)** of the rainbow lorikeet (*Trichoglossus moluccanus*) and projected these onto the tetrahedral color space defined by the receptor sensitivities. Within this color space the distance among points is indicative of their discriminability. Points that are farther apart should be more readily distinguished (Vorobyev et al., 1998; Vorobyev, 2003). We calculated the proportional photon catch of each cone photoreceptor and color space location of these patches for a hypothetical visual system with **(C,F,H)** or without **(B,E,G)** cone oil droplets. The addition of cone oil droplet filtering changes the photon catch of the receptors and shifts the patches toward the vertices of color space. These changes are predicted to increase the volume of color space occupied by the Lorikeet’s plumage patches 37.1% as estimated by the convex hull method of Stoddard and Prum (2008). Lorikeet spectra were adapted from Dalrymple et al. (2015) and cone spectral sensitivities from Wilby et al. (2015). Photo credit: Fir0002/Flagstaffotos.

Vertebrates cannot produce carotenoids *de novo* and must acquire them through the diet. Nonetheless they can metabolize dietary carotenoids to shift their light absorbance spectra (Figures 2, 3A). Within specific cone photoreceptor subtypes, the spectral filtering of the droplets is matched to the sensitivity of the visual pigment by truncating or extending the conjugated system of the carotenoid molecule and modulating the concentrations of the pigments (Figures 2, 3A–C). For example, in the chicken, the red oil droplets (R-type) of the LWS cone are pigmented with the ketocarotenoid astaxanthin, which has a conjugated system of 13 double bonds (Wald and Zussman, 1938; Goldsmith et al., 1984; Toomey et al., 2015). The MWS cone contains an oil droplet (Y-type) pigmented primarily with zeaxanthin, which has a conjugated system of 11 double bonds (Wald and Zussman, 1938; Goldsmith et al., 1984; Toomey et al., 2015). The C-type oil droplet of the SWS2 cone contains galloxanthin, an apocarotenoid that absorbs short-wavelength light and has a conjugated system of eight double bonds (Wald, 1948; Goldsmith et al., 1984; Toomey et al., 2015). Similarly, the oil droplet of the principal member of the double cone (P-type) is pigmented with galloxanthin as well as significant quantities of lutein and zeaxanthin (Goldsmith et al., 1984; Toomey et al., 2015). The SWS1 cone, which is sensitive to ultraviolet and blue light, has an oil droplet (T-type) that contains no measurable pigment (Goldsmith et al., 1984; Toomey et al., 2015). The variable carotenoid composition of the cone oil droplets contributes to a pattern of distinctive autofluorescence among the droplet types (Figure 1B). This autofluorescence facilitates discrimination of P, C and T-type droplets that can appear similar under brightfield illumination (Figures 1A,B; Ohtsuka, 1984; Kram et al., 2010; Toomey et al., 2015). The aforementioned carotenoids are the major constituents of each droplet type in birds, but other carotenoids are present at lower concentrations and likely contribute to the long-pass cut-off filtering function of the droplets (Figure 3A; Toomey et al., 2015). This pattern of cone subtype-specific oil droplet pigmentation is largely conserved among avian species (Goldsmith et al., 1984). However, there is notable variation in the C-type and P-type droplets in some bird species (Coyle et al., 2012; Toomey et al., 2016). Detailed chemical and microspectroscopic analysis of oil droplet composition has not been conducted in most non-avian clades.

The carotenoid pigmentation of cone oil droplets is the product of a multistep process involving the acquisition of precursors from the diet, uptake into the body, transport, chemical modification and selective deposition. In birds, the cone oil droplet pigments astaxanthin and galloxanthin are not typically found in the diet, and radioactive tracing studies indicate that they are produced through the modification of dietary precursors (Figure 2; Goldsmith et al., 1984; Davies, 1986; Bhosale et al., 2007). Studies of the Wisconsin hypoalpha mutant (WHAM) chicken and the white recessive canary, as well as gene expression profiling in developing chicken cones, have implicated high-density lipoproteins (HDL) and scavenger receptor B1 as important mediators of carotenoid transport and uptake to the eye (Connor et al., 2007; Enright et al., 2015; Toomey et al., 2017). β-carotene oxygenase 2 (BCO2) has been implicated as the enzyme that mediates the cleavage of zeaxanthin to form a precursor to galloxanthin, and its expression is localized to a subset of photoreceptors in the chicken retina (Figure 2; Toomey et al., 2016). Recently studies of avian coloration have identified the cytochrome P450 enzyme CYP2J19 as a mediator of ketocarotenoid metabolism underlying red feather coloration (Lopes et al., 2016; Mundy et al., 2016). CYP2J19 is also expressed in a subset of photoreceptors in the developing chicken retina and is hypothesized to convert zeaxanthin into astaxanthin in the red single cone (Lopes et al., 2016; Mundy et al., 2016; Twyman et al., 2016). Thus, the specificity of carotenoid accumulation within the different cone photoreceptor subtypes appears to be mediated by the selective expression of carotenoid metabolizing enzymes within those subtypes. However, there is also evidence for stereoselective carotenoid uptake into the chicken retina, a process which might contribute to differential accumulation between cone subtypes (Schiedt et al., 1991; Schiedt, 1998).

In addition to their role in vision, carotenoids are the pigments that underlie the yellow, orange and red integumentary coloration of a diversity of animals. These colorful displays are an important system for understanding the physiology and evolution of sexually selected traits (Svensson and Wong, 2011). Carotenoids are diet-derived and are implicated in immune system function and antioxidant protection (Svensson and Wong, 2011). Therefore carotenoid-based coloration can reflect aspects of the diet, health and fitness of an individual (Hill et al., 2002; Blount et al., 2003; McGraw and Ardia, 2003; Hamilton et al., 2013). It is tempting to speculate that cone oil droplet pigmentation and, in turn, color vision might be subject to the same environmental and physiological constraints as these colorful ornaments. However, the available evidence does not support this notion. Although manipulations of dietary carotenoid levels and immune system activation can affect the accumulation of carotenoids in oil droplets, the magnitude of these changes are often relatively small, and carotenoids deplete very slowly from the retina, as compared to other tissues (Meyer et al., 1971; Bowmaker et al., 1993; Knott et al., 2010; Toomey and McGraw, 2010, 2011, 2012, 2016; Toomey et al., 2010). Not unexpectedly, the small changes in oil droplet spectral filtering resulting from short-term dietary manipulations have limited impact on visually guided behaviors (Toomey and McGraw, 2011, 2012; Lim and Pike, 2016). Thus, the carotenoid pigmentation of cone oil droplets is relatively stable in the face of environmental and physiological perturbations. This may reflect the importance of droplet spectral filtering in the visual systems of birds.

## Oil Droplet Spectral Filtering Enhances Color Vision

Pigmented cone oil droplets function as long-pass cut-off filters that substantially modify the spectral sensitivity of the photoreceptors (Roaf, 1929; Strother, 1963; Goldsmith et al., 1984). In birds, the pigmented cone oil droplets are essentially transparent at long wavelengths, but absorb nearly all short-wavelength light below a specific cut-off wavelength (*λ*_cut_; Figure 3A). Within each of the single cone photoreceptor subtype, the *λ*_cut_ of the oil droplet is tuned through the accumulation of specific types and concentrations of carotenoids such that the spectral filtering of the droplet complements the sensitivity of the visual pigment (Figures 2, 3A–C). This matched filtering narrows the spectral bandwidth, reduces sensitivity overlap among spectrally adjacent cone subtypes, red-shifts the wavelength of maximum sensitivity (*λ*_max_), and reduces the absolute sensitivity of the cone (Figures 3A–C; Bowmaker and Knowles, 1977; Hart and Vorobyev, 2005). The reduced spectral overlap and narrowing of receptor sensitivity are predicted by computational models of color discrimination and object color space to improve both the discrimination of broad-band spectra and color constancy in changing light conditions (Figures 3D–H; Worthey and Brill, 1986; Vorobyev et al., 1998; Vorobyev, 2003). It is notable that oil droplet filtering can limit the discrimination of narrow-band spectra like monochromatic light, which relies on spectral sensitivity overlap among cone subtypes (Arnold and Neumeyer, 1987). However, such monochromatic spectra are rarely encountered in the natural world.

Recent studies of avian color vision support an important role for cone oil droplet spectral filtering in shaping spectral sensitivity, color discrimination, and color constancy (Goldsmith and Butler, 2003; Lind et al., 2014; Olsson et al., 2015, 2016). For example, behavioral measurements of spectral sensitivity in the budgerigar (*Melopsittacus undulatus*) are consistent with the receptor-noise limited (RNL) model formulated with receptor sensitivities modified by oil droplet spectral filtering (Goldsmith and Butler, 2003; Lind et al., 2014). The RNL model is also a good predictor of the behavioral thresholds of chickens trained to discriminate broad-band spectra (Olsson et al., 2015). Additionally, chickens are more capable of maintaining color constancy and compensating for larger shifts in illumination spectra than species that lack cone oil droplets (Olsson et al., 2016). Although these results support the hypothesized role of oil droplet spectral filtering in color vision, a definitive test requires the coupling of these behavioral approaches with specific manipulations of oil droplet pigmentation. Such tests have proven elusive because diet-based manipulation has limited effects on droplet pigmentation, and behavior may be confounded by a range of other physiological effects of carotenoids. Nonetheless, with our emerging understanding of carotenoid metabolism and new molecular genetic tools, exciting opportunities exist to genetically manipulate droplet pigmentation and dissect its functional role in vision.

Natural variation in ultraviolet sensitivity among bird species provides an opportunity to examine the adaptive function of oil droplet spectral filtering. The spectral sensitivities of avian SWS1 cones fall into one of two classes: those that are maximally sensitive to violet light (*λ*_max_ = 404–426 nm) and those that are maximally sensitive to ultraviolet light (*λ*_max_ = 359–373 nm; Hart, 2001b; Odeen and Hastad, 2003; Ödeen and Håstad, 2013; Hart and Vorobyev, 2005). The bimodality of SWS1 opsin sensitivity is attributable to that fact that a small number of amino acids changes can toggle the *λ*_max_ between violet and ultraviolet (Shi et al., 2001; Altun et al., 2011; Ödeen and Håstad, 2013; Hauser et al., 2014). The amino acid residues that control violet vs. ultraviolet sensitivity of SWS1 opsin are thought to regulate a network of hydrogen bonds around the chromophore, with amino acids that favor deprotonation of the chromophore leading to an ultraviolet shift in sensitivity (Shi et al., 2001; Altun et al., 2011; Hauser et al., 2014). In the course of avian evolution, the *λ*_max_ of the SWS1 cone has switched between violet and ultraviolet multiple times (Ödeen and Håstad, 2013), underscoring the evolutionary accessibility of this shift and suggesting that the selective advantage of either violet or ultraviolet sensitivity depends on the species’ photic niche.

**Figure 4:**
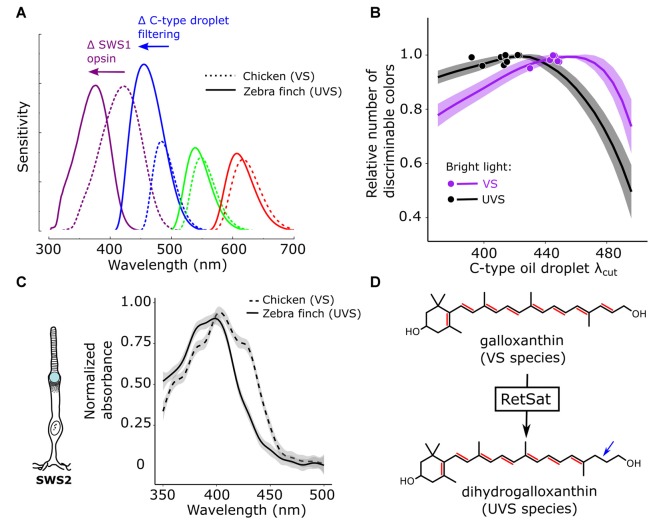
Color discrimination is optimized via co-evolution of opsin and oil droplet spectral tuning. **(A)** Avian visual systems fall in two categories: those with an SWS1 opsin maximally sensitive to violet light (VS, e.g., chicken—dotted lines) and those maximally sensitive to ultraviolet light (UVS, e.g., zebra finch—solid lines). In UVS species, the SWS1 cone sensitivity is short-wavelength shifted, relative to VS species, as the result of amino acid substitutions in the SWS1 visual pigment opsin. In concert with this shift in the SWS1 cone, the sensitivity of the SWS2 cone is also short-wavelength shifted through a change in the carotenoid-based spectral filtering of the C-type oil droplet within the cone. **(B)** These complementary shifts in the sensitivity of the SWS1 and SWS2 cones serve to maintain even spacing of receptor sensitivities across the light spectrum, and computational models of avian color discrimination indicate that these shifts optimize the visual system for color discrimination. Here the receptor noise limited model was used to predict that maximum number of discriminable colors for seven VS species (purple) and 11 UVS species (black). All parameters of the visual system were held constant, while the spectral filtering of the C-type droplet (*λ*_cut_) was varied across a wide range and the number of discriminable colors was determined at each *λ*_cut_ value. The lines represent the mean ± SD model predictions for each visual system and the points represent the actual C-type droplet (*λ*_cut_) for each of the 18 species modeled. Note how relatively long-wavelength shifted C-type filtering maximizes the number of discriminable colors for VS species, relatively short-wavelength shifted filtering maximizes the number of discriminable colors for UVS species, and the measured filtering falls close to the model optima for each visual system. These shifts in spectral filtering of the C-type droplet **(C)** between VS and UVS species result from a change in dominant pigment within the C-type droplet from galloxanthin to dihydrogalloxanthin. **(D)** The enzyme retinol saturase (RETSAT) has been implicated in this conversion. This figure has been adapted from Toomey et al. (2016).

The maximum sensitivities of avian cone photoreceptors are evenly spaced across the light spectrum, a feature that is thought to facilitate color discrimination. In species that have undergone a shift in the *λ*_max_ of the SWS1 cone from violet to ultraviolet, there is a corresponding short-wavelength shift in the *λ*_max_ of the spectrally adjacent SWS2 cone, presumably to maintain even spacing of receptor sensitivities (Figure 4A; Hart, 2001b; Hart and Vorobyev, 2005; Toomey et al., 2016). Surprisingly, the short-wavelength shift of the SWS2 cone is not mediated by changes in the amino acid sequence of the opsin, but rather by modification of the carotenoid content of the cone’s C-type oil droplet (Toomey et al., 2016). In ultraviolet-sensitive species, SWS2 cones convert galloxanthin into dihydrogalloxanthin, which contains one fewer conjugated double bond and therefore has a spectral absorption curve that is short-wavelength shifted by ~22 nm (Figures 4C,D; Toomey et al., 2016). RNL models of avian color space indicate that shifts in C-type droplet spectral filtering serve to maximize the color discrimination capabilities of the respective VS and UVS visual systems in bright-light conditions (Figure 4B; Toomey et al., 2016). Taken together, these results suggest that mechanisms of carotenoid-based spectral filtering in the cone oil droplets have co-evolved with the spectral tuning of the visual pigment opsins to optimize color vision (Toomey et al., 2016).

Accumulating evidence indicates that the spectral filtering of cone oil droplets improves color discrimination in bright light, but this enhancement may come at a cost. The pigmented cone oil droplets block up to 50% of the light reaching the photoreceptor outer segment, substantially reducing overall receptor sensitivity (Bowmaker and Knowles, 1977). Thus, cone oil droplet spectral filtering presents a functional trade-off between fine color discrimination and dim-light sensitivity. For example, budgerigars have pigmented cone oil droplets, are active only in daylight, and lose color vision capabilities at light levels that are ten times greater than the threshold of human color vision (Lind and Kelber, 2009). In contrast, nocturnal owls and penguins are active in light-limited conditions and have depigmented cone oil droplets, suggesting that they trade off enhanced color discrimination to maximize the sensitivity of their receptors (Bowmaker and Martin, 1978, 1985; Gondo and Ando, 1995; Alix et al., 2017). In particular, astaxanthin-pigmented red cone oil droplets are conspicuously absent from the retinas of these dim-light active birds. Recent genomic analyses indicate that *CYP2J19* has been pseudogenized in a range of low-light active bird species, including owls and penguins (Authors’ unpublished results; Emerling, 2018). This result supports CYP2J19 as a key mediator of red oil droplet pigmentation and suggests that this enzyme has no important functions outside of oil droplet pigmentation and integumentary coloration. In general, the depigmentation or loss of cone oil droplets is associated with nocturnal lifestyles and dim-light environments across a broad diversity of vertebrates (see below), indicating that pigmented oil droplets are an adaptation for color vision in bright light.

## Other Functions of Oil Droplet Spectral Filtering

High-energy short-wavelength light is not only potentially damaging to the retina, but also subject to chromatic aberration and tends to scatter as it passes through the optics of the eye, resulting in degradation of spatial resolution (Mandelman and Sivak, 1983; Douglas and Marshall, 1999; Douglas and Cronin, 2016). Many vertebrates avoid these problems by preventing short-wavelength light from reaching the retina with pigmented cornea and lenses, but this filtering precludes the use of these wavelengths for vision (Douglas and Marshall, 1999; Douglas and Cronin, 2016). In species with ocular media that is transparent to short-wavelength light, the specificity of oil droplet spectral filtering among the various cone subtypes allows for short-wavelength sensitivity is some subtypes while protecting outer segments of the other cones from damaging light. Consistent with this protective function, increased carotenoid pigment accumulation in the quail retina is associated with reduced photoreceptor cell death following damaging light exposure (Thomson et al., 2002a,b). It is not clear if this protective effect is the result of droplet spectral filtering or some other action of carotenoids. For example, carotenoid accumulation in the human macula lutea is associated with reduced risk of age-related macular degeneration, and it has been hypothesized that this protective effect might be mediated in part through the antioxidant and anti-inflammatory actions of carotenoids (Seddon et al., 1994; Krinsky et al., 2003; Koushan et al., 2013). Whether cone oil droplet carotenoids play any role in modulating oxidative and inflammatory processes remains a question for future research.

The pigmentation of the oil droplet of double cone photoreceptors in the avian retina also suggests roles for spectral filtering beyond the fine-tuning of receptor sensitivity. Double cone photoreceptors consist of paired principal and accessory cone photoreceptors that are electrically coupled to one another (Smith et al., 1985; Hart, 2001b). Double cones are the most abundant type of cone photoreceptor in the avian retina, and evidence suggests that these receptors mediate achromatic visual processes, including motion detection and brightness discrimination (Sun and Frost, 1997; Campenhausen and Kirschfeld, 1998; Goldsmith and Butler, 2003, 2005; Lind and Kelber, 2011; Lind et al., 2014). These achromatic processes are best served by a receptor with broad spectral sensitivity, and thus there is no apparent benefit to spectral tuning via droplet filtering. Nonetheless, the P-type droplets of double cones are pigmented with short-wavelength absorbing carotenoids. This pigmentation varies within the eyes of a number of bird species with the greatest densities in the ventral retina, a region that typically views the bright celestial hemifield (Hart, 2001b; Knott et al., 2010; Coyle et al., 2012; Toomey et al., 2015). The pigmentation of the double cone oil droplet is also particularly sensitive to environmental light conditions and decreases when birds are maintained in captivity under artificial lighting for extended periods or are reared in dim light (Hart et al., 1998, 1999, 2006; Hart, 2001b). Therefore, it is hypothesized that P-type droplet filtering provides photoprotection and filters scattering short-wavelength light that may compromise the detection of distant objects (Hart, 2001b).

## The Light-Collecting Function of Oil Droplets

The occurrence of colorless (and presumably carotenoid-free) oil droplets in multiple vertebrate clades implies a function aside from spectral filtering. Indeed, even without pigmentation, cone oil droplets are highly refractile (Young and Martin, 1984; Wilby et al., 2015; Wilby and Roberts, 2017). Multiple modeling studies have suggested that oil droplets collect light and focus it into the outer segment, enhancing light capture and thereby increasing cone sensitivity (Baylor and Fettiplace, 1975; Govardovskii et al., 1981; Ives et al., 1983; Young and Martin, 1984; Stavenga and Wilts, 2014). Colorless oil droplets are frequently found in the cones of nocturnal species, as well as in the cones of species intermittently active in dim light (e.g., deep-diving penguins; Bowmaker and Martin, 1978, 1984; Gondo and Ando, 1995; Alix et al., 2017). Even strongly diurnal species with heavily pigmented oil droplets possess SWS1 cones with colorless oil droplets (Goldsmith et al., 1984; Toomey et al., 2015), and in some species, depigmentation of all oil droplet types occurs in the central retina, in areas mediating high-acuity vision (Walls and Judd, 1933; Hart, 2001a). The presence of colorless oil droplets in such a wide range of species offers *prima facie* evidence for a functional role besides spectral filtering.

**Figure 5:**
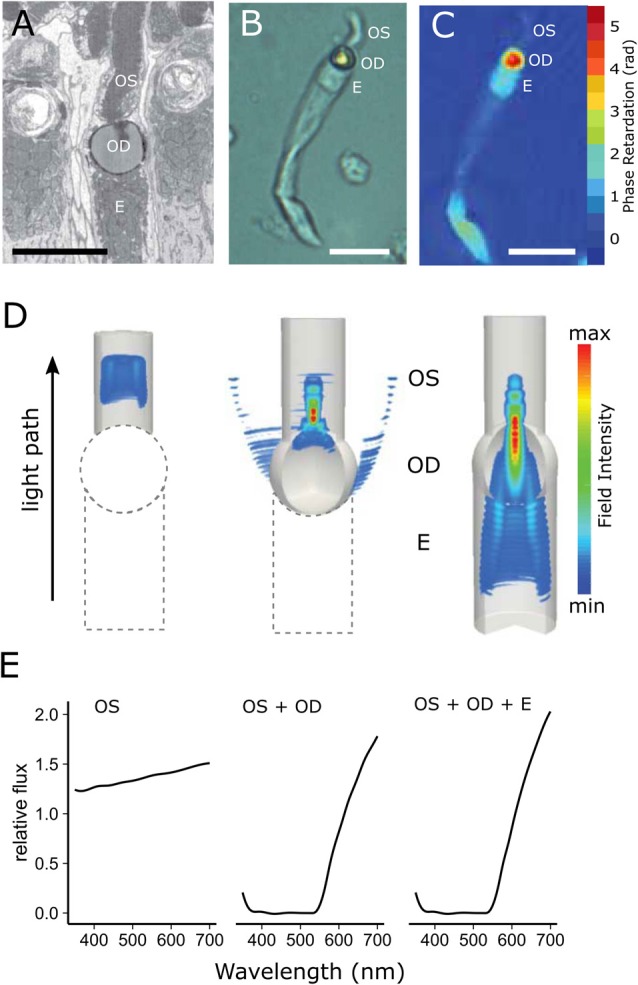
Cone oil droplets enhance light capture by the outer segment. **(A)** An electron micrograph of a chicken cone photoreceptor shows that the cone oil droplet (OD) is located at the inner and outer segment (OS) interface immediately adjacent to the ellipsoid (E). **(B)** An isolated MWS single cone from the chicken under bright field illumination. **(C)** The same cell imaged with digital holographic microscopy to map the phase retardation of light through different portions of the cell. Phase retardation correlates with the refractive index of the various subcellular compartments. **(D)** Finite-difference time-domain (FDTD) simulations of light (550 nm) propagation through three configurations of the LWS cone photoreceptor (left = outer segment alone; middle = outer segment + oil droplet; right outer segment + oil droplet + ellipsoid). Note that there is considerable scattering of light at the droplet interface (middle panel), but that when the droplet is combined with the ellipsoid, scatter is reduced and flux to the outer segment is enhanced (right panel). **(E)** The predicted flux of light to the outer segment for the three configurations of the photoreceptor. The addition of the oil droplet dramatically reduces flux due to the spectral absorbance of the droplet at shorter wavelengths and light scattering across much of the spectrum, but modestly enhances flux at longer wavelengths. This figure has been modified with permission from Wilby et al. (2015).

Recent studies confirm that oil droplets can indeed enhance light capture by the outer segment, but that this effect is more modest than previously thought and is restricted to non-pigmented droplets (Stavenga and Wilts, 2014; Wilby et al., 2015; Wilby and Roberts, 2017). Using finite-difference time-domain (FDTD) optical modeling, Wilby et al. (2015) found that several factors can influence the light-collecting properties of oil droplets, including: the refractive index of the droplet, the dimensions of the droplet and the outer segment, and the presence and refractive index of the ellipsoid (Figure 5; Wilby et al., 2015; Wilby and Roberts, 2017). An increase in the refractive index of the oil droplet (due to an increase in the concentration of carotenoids) results in a decrease in light capture, likely on account of increased light reflection at the inner segment-droplet interface caused by the abrupt transition in refractive index at this juncture (Figure 5; Wilby et al., 2015). Wilby et al. (2015) also found that the presence of an ellipsoid with a refractive index intermediate between that of the oil droplet and inner segment cytoplasm partially mitigates the loss of light at the inner segment-droplet interface, thereby enhancing light delivery to the outer segment (Figure 5). These modeling approaches also suggest that increasing oil droplet diameter and decreasing outer segment length results in increased light capture (Wilby and Roberts, 2017). Modeling studies also suggest that oil droplets focus light on the central portion of the outer segment, but the potential function of such focusing is unknown (Wilby et al., 2015). Another possible optical function of oil droplets is as modulators of the angular sensitivity of photoreceptors (Baylor and Fettiplace, 1975; Wilby et al., 2015).

These findings suggest that the presence of carotenoids decreases light capture both by increasing the refractive index of the droplet (a wavelength-independent effect) and by absorbing light below a cut-off wavelength that is determined by the type and concentration of carotenoids present. The widespread occurrence of pigmented oil droplets in diurnal species implies that the benefits of improved color vision conferred by oil droplets outweigh the negative effects on light capture and sensitivity. Conversely, the presence of colorless oil droplets in many dim-light species indicates that the adverse effects of pigmentation on light capture are sufficiently strong to select against carotenoid deposition in such species. The complete loss of oil droplets in certain dim-light clades (e.g., snakes, eutherian mammals) suggests that the light-collecting function may be of marginal utility in low-light environments (Walls, 1942). In further support of this notion, oil droplets have only rarely been observed in rods (Walls, 1942; Appudurai et al., 2016). Spatial summation and convergence of multiple rod signals onto a single retinal ganglion cell may suffice to enhance sensitivity in dim environments, thus rendering oil droplets superfluous.

## Phylogenetic Distribution of Oil Droplets

**Figure 6:**
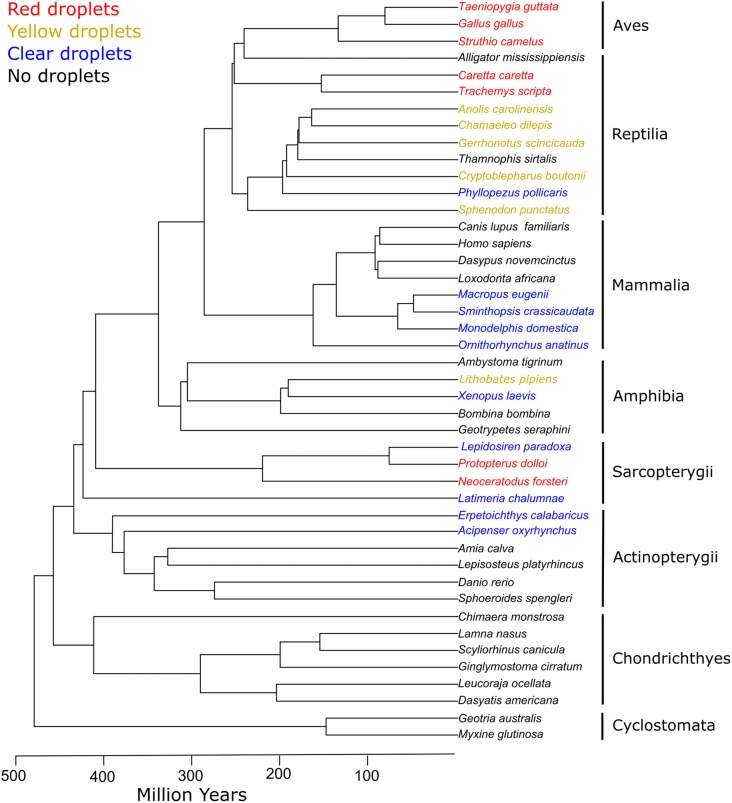
Cone oil droplets are widely distributed among vertebrate clades. The presence of cone oil droplets in each species is indicated by the color of the branch tip label. Black indicates that oil droplets are not present in that species. Blue indicates the presence of colorless oil droplets only. Yellow indicates the presence of yellow and colorless droplets. Red denotes the presence of red, yellow, and colorless droplets (except for Sarcopterygii which lack yellow droplets). Tree topology and timescales are based on Irisarri et al. (2017).

Oil droplets occur in the photoreceptors of a diversity of vertebrates from fish to mammals, indicating that this organelle appeared early in vertebrate evolution (Figure 6; Walls, 1942; Robinson, 1994). The variable occurrence and properties of cone oil droplets among taxa are broadly consistent with the trade-offs between bright-light color discrimination and dim-light sensitivity discussed above. Here we briefly survey the distribution and morphology of the oil droplets within each of the vertebrate classes, in relation to their phylogeny and visual ecology.

### Actinopterygii

Cone oil droplets have never been identified in Cyclostomata (lamprey and hagfish) or Chondrichthyes (sharks, rays, skates and chimeras), and their occurrence in ray-finned fishes (Actinopterygii) is limited to a small number of species in the orders Polypteriformes and Acipenseriformes, taxa that diverged very early in the evolution of Actinopterygii (Ali and Anctil, 1976; Sillman and Dahlin, 2004). All sturgeon and paddlefish species (Acipenseriformes) examined to date have colorless oil droplets within their cone photoreceptors (Ali and Anctil, 1976; Sillman et al., 1990, 1995, 1999, 2005; Govardovskii et al., 1992). Reedfish (*Erpetoichthys calabaricus*; Polypteriformes) also have colorless oil droplets within their single cones and in the principal member of their double cones (Ali and Anctil, 1976). Cone oil droplets are absent from the retinas of actinopterygian orders that diverged later in the evolutionary history of this class (Walls, 1942; Ali and Anctil, 1976). These orders represent some of the most diverse vertebrate taxa with widely varied life histories and ecologies, so the lack of cone oil droplets cannot be readily linked to any common aspect of their current visual ecology. Rather, it has been hypothesized that oil droplets were lost in a nocturnal or dim-light adapted ancestor and have not subsequently reemerged in the course of fish evolution (Walls, 1942; Robinson, 1994). However, as discussed above, some taxa within this group have evolved ellipsosome structures that may be functionally convergent with cone oil droplets.

### Sarcopterygii

The lobe-finned fishes (Sarcopterygii) are sister taxa to the tetrapods and diverged from this lineage more than 400 Mya (Figure 6; Irisarri et al., 2017). All of the lobe-finned fishes examined to date have cone oil droplets in their retinas, indicating that these structures are an ancestral trait in the tetrapod lineage and likely originated sometime before the Actinopterygii-Sarcopterygii split ~449 Mya (Walls, 1942; Robinson, 1994; Irisarri et al., 2017). The deep-sea dwelling coelacanth (*Latimeria chalumnae*) inhabits a dim-light environment and has a rod-dominated retina with a single class of cone photoreceptors that contains a colorless oil droplet (Ali and Anctil, 1976; Yokoyama et al., 1999). The Australian lungfish (*Neoceratodus forsteri*), a species active in bright light, has four cone photoreceptor subtypes (Hart et al., 2008). The LWS cones of this species contain a red oil droplet, whereas the MWS cone lacks an oil droplet and has instead a yellow-pigmented ellipsoid (Bailes et al., 2006; Hart et al., 2008). The remaining cone subtypes contain colorless oil droplets (Bailes et al., 2006; Hart et al., 2008). The chemical composition of these oil droplets has not been investigated, but the spectral properties of the red droplets are similar to the astaxanthin-pigmented droplets of turtles and birds. The spotted African lungfish (*Protopterus dolloi*) has a single cone photoreceptor containing a red oil droplet, another cone subtype without an oil droplet, and a colorless oil droplet within their rod photoreceptors (Appudurai et al., 2016). The rod and cone photoreceptors of the South American lungfish (*Lepidosiren paradoxa*) both contain colorless oil droplets, and it is speculated that this species may be less dependent on color vision than other lungfishes (Ali and Anctil, 1973; Appudurai et al., 2016). The presence of oil droplets in the rod photoreceptors is surprising, given their absence from the rods of the majority of other taxa. However, the receptors subtypes have only been superficially characterized in the African and South American lungfishes, and these observations warrant further investigation.

### Amphibia

Among the amphibians, oil droplets have been described in the cone photoreceptors of some species of frogs (Anura), but are absent from the photoreceptors of salamanders (Caudata) and caecilians (Gymnophonia; Walls, 1942). The distribution of cone oil droplets among the Anurans is complex, and the retinas of less than half of the families in this diverse order have been characterized. The only comparative study of this clade was completed by Hailman (1976), and he noted the presence of cone oil droplets in the retinas of Dendrobatid, Rhacophorid, Ranid, Microhylid and Pipid species and their absence from the retinas of other families including Bufonidae and Hylidae. Detailed accounts of the photoreceptors of the African clawed frog (*Xenopus laevis*) indicate that cone oil droplets are found within the principal member of the double cone and all three classes of single cone photoreceptors (Rohlich and Szel, 2000). These droplets are colorless and have similar refractive indices, suggesting that droplet composition does not differ greatly among the cone subtypes (Wilby and Roberts, 2017). Similarly, the cone oil droplets of the strawberry poison frog (*Dendrobates pumilio*) are colorless with no significant light absorbance across the visible spectrum (350–700 nm; Siddiqi et al., 2004). The cone oil droplets of the northern leopard frog (*Lithobates pipens*) and green frog (*Lithobates clamitans*) are described as having yellow pigmentation; however, the spectral absorbance and chemical composition of these droplets have not been measured in detail (Liebman and Entine, 1968; Hailman, 1976; Hisatomi et al., 1998). Overall, the limited taxonomic coverage and paucity of detailed characterizations makes it difficult to link oil droplet occurrence and morphology to specific aspects of visual ecology and evolutionary history among the Anurans.

### Reptilia

Oil droplets are present in the cones of turtles and tuatara (*Sphenodon punctatus*), which diverged early in the evolutionary history of the Diapsids, indicating that cone oil droplets are an ancestral trait in the reptile lineage (Figure 6; Walls, 1934, 1942; Robinson, 1994; Meyer-Rochow et al., 2005). All species of turtles examined to date have oil droplets of varying colors within their cone photoreceptors with most detailed studies having been made in the red-eared slider (*Trachemys scripta elegans*; Granda and Haden, 1970; Liebman and Granda, 1971; Neumeyer and Jäger, 1985; Loew and Govardovskii, 2001). Similar to the avian retina, the red-eared slider has five different cone photoreceptor subtypes that each contains a different colored oil droplets. The LWS single cone contains a red or orange droplet, the MWS cone a yellow droplet, the SWS2 cone a colorless, ultraviolet-absorbing droplet, and the SWS1 cone a colorless, carotenoid-free droplet. The LWS-containing double cone contains a pale green oil droplet in its principal member (Loew and Govardovskii, 2001). Similar to the avian retina described above, the cone oil droplets of the turtle retina are pigmented with carotenoids. For example, the red and yellow oil droplets of the red-eared slider have absorbance spectra consistent with astaxanthin and zeaxanthin, respectively (Liebman and Granda, 1975). In contrast, the ultraviolet-absorbing apocarotenoids and yellow carotenes in turtle cone oil droplets appear distinct from those found in the avian retina and warrant further investigation (Liebman and Granda, 1975; Loew and Govardovskii, 2001). The tuatara retina reportedly contains cone photoreceptors with green oil droplets, but detailed measurements of droplet spectral absorbance and composition have not been made (Walls, 1934, 1942; Meyer-Rochow et al., 2005). In contrast to the tuatara and turtles, oil droplets are absent from the photoreceptors of similarly ancient Crocydilians (Walls, 1942; Sillman et al., 1991).

The majority of lizards examined to date have colorless, pale green and/or yellow oil droplets within their cone photoreceptors. Red oil droplets have never been observed in this group. For example, the Carolina anole (*Anolis carolinensis*) has a cone-dominated retina typical of diurnal lizards with five classes of cones containing differently colored oil droplets: the principal member of the double cones contains a greenish oil droplet, the LWS single cone contains a yellow droplet, the MWS cone a greenish droplet, and the SWS1 and SWS2 cones contain clear droplets (Provencio et al., 1992; Loew et al., 2002). Chamaeleonidae species are reported to have a similar complement of cone receptor subtypes with colored oil droplets (Bowmaker et al., 2005). The chemical composition of diurnal lizard droplets has not been investigated in detail. However, the spectral properties of the cone oil droplets are consistent with xanthophyll carotenoids in the yellow droplets and apocarotenoids (such as galloxanthin) in the pale greenish droplets (Barbour et al., 2002; Bowmaker et al., 2005).

The Gekkonidae offer valuable insights into droplet evolution and function because members of this family have divergent diurnal and nocturnal lifestyles. The diurnal *Gonatodes* and *Phelsuma* genera retain colorless oil droplets in their cone photoreceptors, while droplets have been lost in all other nocturnal gecko genera suggesting that the loss of droplets is an adaption for dim-light conditions (Röll, 2000, 2001). Oil droplets are also absent from the photoreceptors of snakes (Ophidia), and this loss has been attributed to the fossorial origins of this clade that involved a reduction in the complexity of the eye and adaptation for dim light (Walls, 1934, 1942; Wong, 1989; Sillman et al., 1997). While some snakes have returned to a diurnal lifestyle, none appear to have re-evolved cone oil droplets. However, ellipsoidal microdroplets have been observed in the common garter snake (*Thamnophis sirtalis*) and are hypothesized to play an optical role similar to oil droplets (Bossomaier et al., 1989; Wong, 1989).

### Aves

Cone oil droplets are an omnipresent feature of the avian retina and are richly pigmented in diurnal species (Walls, 1942; Goldsmith et al., 1984; Hart, 2001b). The majority of birds have five distinct cone photoreceptors that each contain a different colored oil droplet. In the chicken for example, the principal member of the principal member of the double cone contains a pale green droplet, the LWS single cone contains a brilliant red droplet, the MWS cone a yellow droplet, the SWS2 cone a very pale green droplet, and the SWS1 cone a colorless, transparent droplet (Figure 1C; Bowmaker and Knowles, 1977; Goldsmith et al., 1984; Bowmaker et al., 1997). As described in preceding sections, these droplets are pigmented with various carotenoids and play an important role in avian color vision (Goldsmith et al., 1984; Vorobyev et al., 1998; Vorobyev, 2003; Toomey et al., 2015, 2016; Wilby et al., 2015).

Although all birds have cone oil droplets, the pigmentation of the droplets varies with the visual ecology of the species. Nocturnal or dim-light active bird species have very pale or colorless cone oil droplets. The nocturnal tawny owl (*Strix aluco*), snowy owl (*Bubo scandiacus*), Ural owl (*Strix uralensis*) and tawny frogmouth (*Podargus strigoides*) are all noted to have depigmented oil droplets with the red cone droplet being conspicuously absent (Bowmaker and Martin, 1978; Gondo and Ando, 1995; Hart et al., 2006; Alix et al., 2017). Similarly, the cone oil droplets of King (*Aptenodytes patagonicus*) and Humboldt (*Spheniscus humboldti*) penguins are relatively depigmented, and these species also lack red cone oil droplets (Bowmaker and Martin, 1985; Gondo and Ando, 1995). These marine foraging birds encounter dim-light conditions while foraging at depths of more than 300 m, and depigmentation of the droplets is likely an adaptation to increase the absolute sensitivity of the receptors. Similarly, several diurnal bird species have been shown to have oil droplets that are selectively depigmented in the central, high-acuity region of the retina, possibly to enhance spatial resolution (Walls and Judd, 1933; Hart, 2001a).

### Mammalia

During the age of dinosaurs, the ancestors of modern mammals are thought to have passed through a long period of nocturnality (the “nocturnal bottleneck”). Many features of their visual systems, including the morphology and occurrence of cone oil droplets, reflect this evolutionary history (Walls, 1942; Jacobs, 1993, 2009). Cone oil droplets are entirely absent from retinas of eutherian mammals, and only colorless droplets are present in the cone photoreceptors of some monotremes and marsupials (Figure 6). The duck-billed platypus (*Ornithorhyncus anatinus*) has a rod-dominated retina, but there are at least three cone photoreceptor subtypes that each contain a colorless oil droplet (Zeiss et al., 2011). Cone oil droplets are not a universal feature of the monotreme retina, however, as they are absent from the cones of the echidna (*Tachyglossus aculeatus*, Young and Pettigrew, 1991). All marsupial species examined to date have rod-dominated retinas with single and double cone photoreceptors that contain colorless oil droplets (O’Day, 1936; Walls, 1939, 1942; Braekevelt, 1973; Ahnelt et al., 1995; Arrese et al., 1999, 2005; Hemmi and Grünert, 1999; Hemmi et al., 2000; Vlahos et al., 2014).

Eutherian mammals (including mice and humans) do not have cone oil droplets within their photoreceptors. Nonetheless, some eutherian mammals have adaptations that are functionally convergent with oil droplets. For example, diurnal primates, sciurid rodents, meerkats and tree shrews have kynurenine-derived pigments in their lenses which filter short-wavelength light, a function ascribed to pigmented cone oil droplets in other taxa (Zigman and Paxhia, 1988; Douglas and Jeffery, 2014; Lyons et al., 2014). The macula lutea of the diurnal primate retina derives its name from its yellow coloration that is the result of the accumulation of xanthophyll carotenoid pigments (Snodderly et al., 1984a,b). Like the colorful cone oil droplets of birds and turtles, this macular pigment functions as a spectral filter. However, the carotenoids of the macula lutea accumulate in the cell membrane of the photoreceptor axons (Henle fibers; Bone and Landrum, 1984; Snodderly et al., 1984a,b; Hammond et al., 2013). Lastly, megamitochondria have been reported in the ellipsoids of tree shrews (*Tupaia belangeri*) and shrews (*Sorex sp*.) and may serve an optical function similar to oil droplets (Nag and Bhattacharjee, 1995; Knabe et al., 1997; Flamarique and Hárosi, 2000; Lluch et al., 2003).

## Conclusion

Cone oil droplets have a wide taxonomic distribution, and there is growing evidence that they play an important role in the function of the visual system. Nonetheless, these fascinating organelles have received comparatively limited attention from the modern vision science community. There now are tremendous opportunities to apply cutting-edge molecular, biochemical and optical approaches to unravel the development and function of cone oil droplets and thereby expand our understanding of comparative vertebrate visual function. For example, the developmental and organellar origin of the oil droplets remains unknown, and understanding this process could offer general insights into the mechanisms of lipid metabolism. The structure and composition of oil droplets, particularly in non-avian species, remains incompletely defined but could be readily elucidated with current chromatographic, proteomic and mass spectral analytic approaches. The pigmentation of the cone oil droplets of birds and turtles offers a model system that has begun to yield insight into vertebrate carotenoid physiology, but many questions remain about how these pigments are transported and metabolized. Recent measurements and modeling of the optical properties of cone oil droplets support their hypothesized role in determining the flux of light through the receptor. These models offer a rich set of predictions that await empirical testing. Oil droplets are associated with diurnal lifestyles that rely on color vision and are often lost in nocturnal lineages. This pattern suggests that oil droplets may present a functional trade-off between bright-light color vision and dim-light sensitivity. Definitive tests of these hypotheses will require coupling genetic manipulations of oil droplet pigmentation and development with detailed behavioral measures of visual function.

## Author Contributions

MBT and JCC conceived and drafted the manuscript. All authors read and approved the final manuscript.

## Conflict of Interest Statement

The authors declare that the research was conducted in the absence of any commercial or financial relationships that could be construed as a potential conflict of interest.

## References

[B1] Ahnelt P. K. Hokoç J. N. Röhlich P. (1995). Photoreceptors in a primitive mammal, the South American opossum, *Didelphis marsupialis aurita*: characterization with anti-opsin immunolabeling. Vis. Neurosci. 12, 793–804. 10.1017/s0952523800009366 8924404

[B2] Ali M. A. Anctil M. (1973). Retina of the South American lungfish, *Lepidosiren paradoxa* Fitzinger. Can. J. Zool. 51, 969–972. 10.1139/z73-140 4779819

[B3] Ali M. A. Anctil M. (1976). Retinas of Fishes: An Atlas. Berlin: Springer.

[B4] Alix B. Segovia Y. Garcia M. (2017). The structure of the retina of the Eurasian Eagle-owl and its relation to lifestyle. Avian Biol. Res. 10, 36–44. 10.3184/175815617x14799886573147

[B5] Altun A. Morokuma K. Yokoyama S. (2011). H-bond network around retinal regulates the evolution of ultraviolet and violet vision. ACS Chem. Biol. 6, 775–780. 10.1021/cb200100f 21650174 PMC3158842

[B6] Appudurai A. M. Hart N. S. Zurr I. Collin S. P. (2016). Morphology, characterization and distribution of retinal photoreceptors in the south american (*Lepidosiren paradoxa*) and spotted african (*Protopterus dolloi*) lungfishes. Front. Ecol. Evol. 4:78 10.3389/fevo.2016.00078

[B7] Arnold K. Neumeyer C. (1987). Wavelength discrimination in the turtle *Pseudemys scripta elegans*. Vision Res. 27, 1501–1511. 10.1016/0042-6989(87)90159-3 3445484

[B8] Arrese C. Dunlop S. A. Harman A. M. Braekevelt C. R. Ross W. M. Shand J.. (1999). Retinal structure and visual acuity in a polyprotodont marsupial, the fat-tailed dunnart (*Sminthopsis crassicaudata*). Brain Behav. Evol. 53, 111–126. 10.1159/000006588 10085478

[B9] Arrese C. A. Oddy A. Y. Runham P. B. Hart N. S. Shand J. Hunt D. M.. (2005). Cone topography and spectral sensitivity in two potentially trichromatic marsupials, the quokka (*Setonix brachyurus*) and quenda (*Isoodon obesulus*). Proc. Biol. Sci. 272, 791–796. 10.1098/rspb.2004.3009 15888411 PMC1599861

[B10] Bailes H. J. Robinson S. R. Trezise A. E. Collin S. P. (2006). Morphology, characterization, and distribution of retinal photoreceptors in the Australian lungfish *Neoceratodus forsteri* (Krefft, 1870). J. Comp. Neurol. 494, 381–397. 10.1002/cne.20809 16320259

[B11] Barbour H. R. Archer M. A. Hart N. S. Thomas N. Dunlop S. A. Beazley L. D.. (2002). Retinal characteristics of the ornate dragon lizard, *Ctenophorus ornatus*. J. Comp. Neurol. 450, 334–344. 10.1002/cne.10308 12209847

[B12] Baylor D. A. Fettiplace R. (1975). Light path and photon capture in turtle photoreceptors. J. Physiol. 248, 433–464. 10.1113/jphysiol.1975.sp010983 1151792 PMC1309531

[B13] Bhosale P. Serban B. Zhao D. Y. Bernstein P. S. (2007). Identification and metabolic transformations of carotenoids in ocular tissues of the Japanese quail *Coturnix japonica*. Biochemistry 46, 9050–9057. 10.1021/bi700558f 17630780 PMC2531157

[B14] Blount J. D. Metcalfe N. B. Birkhead T. R. Surai P. F. (2003). Carotenoid modulation of immune function and sexual attractiveness in zebra finches. Science 300, 125–127. 10.1126/science.1082142 12677066

[B15] Bone R. Landrum J. (1984). Macular pigment in Henle fiber membranes: a model for Haidinger’s brushes. Vision Res. 24, 103–108. 10.1016/0042-6989(84)90094-4 6546825

[B16] Bossomaier T. R. Wong R. O. Snyder A. W. (1989). Stiles-Crawford effect in garter snake. Vision Res. 29, 741–746. 10.1016/0042-6989(89)90036-9 2626831

[B21] Bowmaker J. K. Heath L. A. Wilkie S. E. Hunt D. M. (1997). Visual pigments and oil droplets from six classes of photoreceptor in the retinas of birds. Vision Res. 37, 2183–2194. 10.1016/s0042-6989(97)00026-6 9578901

[B17] Bowmaker J. K. Knowles A. (1977). The visual pigments and oil droplets of the chicken retina. Vision Res. 17, 755–764. 10.1016/0042-6989(77)90117-1 898682

[B22] Bowmaker J. K. Kovach J. K. Whitmore A. V. Loew E. R. (1993). Visual pigments and oil droplets in genetically manipulated and carotenoid deprived quail: a microspectrophotometric study. Vision Res. 33, 571–578. 10.1016/0042-6989(93)90180-5 8351832

[B23] Bowmaker J. K. Loew E. R. Ott M. (2005). The cone photoreceptors and visual pigments of chameleons. J. Comp. Physiol. A Neuroethol. Sens. Neural Behav. Physiol. 191, 925–932. 10.1007/s00359-005-0014-4 16025336

[B18] Bowmaker J. K. Martin G. R. (1978). Visual pigments and colour vision in a nocturnal bird, *Strix aluco* (Tawny owl). Vision Res. 18, 1125–1130. 10.1016/0042-6989(78)90095-0 716232

[B19] Bowmaker J. K. Martin G. R. (1984). Color-vision in the penguin, *Spheniscus humboldti*: a microspectrophotometric Study. Vision Res. 24, 1702–1702. 10.1016/0042-6989(84)90359-6

[B20] Bowmaker J. K. Martin G. R. (1985). Visual pigments and oil droplets in the penguin, *Spheniscus humboldti*. J. Comp. Physiol. A Neuroethol. Sens. Neural Behav. Physiol. 156, 71–77. 10.1007/BF00610668

[B24] Braekevelt C. R. (1973). Fine structure of the retinal pigment epithelium and photoreceptor cells of an Australian marsupial *Setonix brachyurus*. Can. J. Zool. 51, 1093–1100. 10.1139/z73-159 4791801

[B25] Campenhausen M. V. Kirschfeld K. (1998). Spectral sensitivity of the accessory optic system of the pigeon. J. Comp. Physiol. A Neuroethol. Sens. Neural Behav. Physiol. 183, 1–6. 10.1007/s003590050229

[B26] Chábera P. Fuciman M. Hríbek P. Polívka T. (2009). Effect of carotenoid structure on excited-state dynamics of carbonyl carotenoids. Phys. Chem. Chem. Phys. 11, 8795–8803. 10.1039/b909924g 20449025

[B27] Connor W. E. Duell P. B. Kean R. Wang Y. M. (2007). The prime role of HDL to transport lutein into the retina: Evidence from HDL-Deficient WHAM chicks having a mutant ABCA1 transporter. Invest. Ophthalmol. Vis. Sci. 48, 4226–4231. 10.1167/iovs.06-1275 17724211

[B28] Coyle B. J. Hart N. S. Carleton K. L. Borgia G. (2012). Limited variation in visual sensitivity among bowerbird species suggests that there is no link between spectral tuning and variation in display colouration. J. Exp. Biol. 215, 1090–1105. 10.1242/jeb.062224 22399654

[B29] Dalrymple R. L. Hui F. K. C. Flores-Moreno H. Kemp D. J. Moles A. T. (2015). Roses are red, violets are blue—so how much replication should you do? An assessment of variation in the colour of flowers and birds. Biol. J. Linn. Soc. 114, 69–81. 10.1111/bij.12402

[B30] Davies B. W. (1986). Xanthophylls as Metabolic Precursors. [Dissertation] Aberystwyth, Wales: University College of Wales.

[B31] Donner K. O. (1960). “On the effect of the coloured oil droplets in the spectral sensitivity of the avian retina,” in Proceedings of the 12th International Ornithological Congress (Helsinki, 1958), 167–172.

[B32] Douglas R. H. Cronin T. W. (2016). “Visual matched filtering in vertebrates,” in The Ecology of Animal Senses, eds Von Der Emde G. Warrant E. (Cham: Springer International Publishing), 169–203.

[B33] Douglas R. H. Jeffery G. (2014). The spectral transmission of ocular media suggests ultraviolet sensitivity is widespread among mammals. Proc. Biol. Sci. 281:20132995. 10.1098/rspb.2013.2995 24552839 PMC4027392

[B34] Douglas R. H. Marshall N. J. (1999). “A review of vertebrate and invertebrate ocular filters,” in Adaptive Mechanisms in the Ecology of Vision, eds Archer S. N. Djamgoz M. B. A. Loew E. R. Partridge J. C. Vallerga S. (Dordrecht, Netherlands: Kluwer Academic Publishers), 95–162.

[B35] Emerling C. A. (2018). Independent pseudogenization of *CYP2J19* in penguins, owls and kiwis implicates gene in red carotenoid synthesis. Mol. Phylogenet. Evol. 118, 47–53. 10.1016/j.ympev.2017.09.016 28943375

[B36] Enright J. M. Lawrence K. A. Hadzic T. Corbo J. C. (2015). Transcriptome profiling of developing photoreceptor subtypes reveals candidate genes involved in avian photoreceptor diversification. J. Comp. Neurol. 523, 649–668. 10.1002/cne.23702 25349106 PMC4367231

[B37] Flamarique I. N. Hárosi F. I. (2000). Photoreceptors, visual pigments and ellipsosomes in the killifish, *Fundulus heteroclitus*: a microspectrophotometric and histological study. Vis. Neurosci. 17, 403–420. 10.1017/s0952523800173080 10910108

[B38] Goldsmith T. H. Butler B. K. (2003). The roles of receptor noise and cone oil droplets in the photopic spectral sensitivity of the budgerigar, *Melopsittacus undulatus*. J. Comp. Physiol. A Neuroethol. Sens. Neural Behav. Physiol. 189, 135–142. 10.1007/s00359-002-0385-8 12607042

[B39] Goldsmith T. H. Butler B. K. (2005). Color vision of the budgerigar (*Melopsittacus undulatus*): hue matches, tetrachromacy, and intensity discrimination. J. Comp. Physiol. A Neuroethol. Sens. Neural Behav. Physiol. 191, 933–951. 10.1007/s00359-005-0024-2 16086150

[B40] Goldsmith T. H. Collins J. S. Licht S. (1984). The cone oil droplets of avian retinas. Vision Res. 24, 1661–1671. 10.1016/0042-6989(84)90324-9 6533991

[B41] Gondo M. Ando H. (1995). Comparative and histophysiological study of oil droplets in the avian retina. Kobe J. Med. Sci. 41, 127–139. 8904163

[B42] Goodwin T. W. (1984). The Biochemistry of the Carotenoids. New York, NY: Chapman and Hall.

[B43] Govardovskii V. I. Golovanevskii E. I. Zueva L. V. Vasil’eva I. L. (1981). Role of cellular organoids in photoreceptor optics (studies on microwave models). Zh. Evol. Biokhim. Fiziol. 17, 492–497, (in Russian). 7293565

[B44] Govardovskii V. I. Röhlich P. Szél A. Zueva L. V. (1992). Immunocytochemical reactivity of rod and cone visual pigments in the sturgeon retina. Vis. Neurosci. 8, 531–537. 10.1017/s0952523800005629 1534023

[B45] Granda A. M. Haden K. W. (1970). Retinal oil globule counts and distributions in two species of turtles: *Pseudemys scripta elegans* (Wied) and *Chelonia mydas mydas* (Linnaeus). Vision Res. 10, 79–84. 10.1016/0042-6989(70)90064-7 5435015

[B46] Hailman J. F. (1964). Coding of the colour preference of the gull chick. Nature 204:710 10.1038/204710a0

[B47] Hailman J. P. (1976). Oildroplets in the eyes of adult anuran amphibians: a comparative survey. J. Morphol. 148, 453–468. 10.1002/jmor.1051480404 180296

[B48] Hamilton D. G. Whiting M. J. Pryke S. R. (2013). Fiery frills: carotenoid-based coloration predicts contest success in frillneck lizards. Behav. Ecol. 24, 1138–1149. 10.1093/beheco/art041

[B49] Hammond B. R. Jr. Fletcher L. M. Elliott J. G. (2013). Glare disability, photostress recovery, and chromatic contrast: relation to macular pigment and serum lutein and zeaxanthin. Invest. Ophthalmol. Vis. Sci. 54, 476–481. 10.1167/iovs.12-10411 23211814

[B50] Hannover A. (1840). Über die Netzhaut und ihre Gehirnsubstanz bei Wirbeltieren mit Ausnahme des Menschen. Müllers Arch. Anat. Physiol. 320–345.

[B51] Hart N. Partridge J. Cuthill I. I. (1998). Visual pigments, oil droplets and cone photoreceptor distribution in the european starling (*Sturnus vulgaris*). J. Exp. Biol. 201, 1433–1446. 9547323 10.1242/jeb.201.9.1433

[B52] Hart N. S. (2001a). Variations in cone photoreceptor abundance and the visual ecology of birds. J. Comp. Physiol. A 187, 685–697. 10.1007/s00359-001-0240-3 11778831

[B53] Hart N. S. (2001b). The visual ecology of avian photoreceptors. Prog. Retin Eye Res. 20, 675–703. 10.1016/s1350-9462(01)00009-x 11470455

[B55] Hart N. S. Bailes H. J. Vorobyev M. Marshall N. J. Collin S. P. (2008). Visual ecology of the Australian lungfish (*Neoceratodus forsteri*). BMC Ecol. 8:21. 10.1186/1472-6785-8-21 19091135 PMC2639370

[B56] Hart N. S. Lisney T. J. Collin S. P. (2006). Cone photoreceptor oil droplet pigmentation is affected by ambient light intensity. J. Exp. Biol. 209, 4776–4787. 10.1242/jeb.02568 17114410

[B57] Hart N. S. Partridge J. C. Cuthill I. C. (1999). Visual pigments, cone oil droplets, ocular media and predicted spectral sensitivity in the domestic turkey (*Meleagris gallopavo*). Vision Res. 39, 3321–3328. 10.1016/s0042-6989(99)00071-1 10615498

[B54] Hart N. S. Vorobyev M. (2005). Modelling oil droplet absorption spectra and spectral sensitivities of bird cone photoreceptors. J. Comp. Physiol. A Neuroethol. Sens. Neural Behav. Physiol. 191, 381–392. 10.1007/s00359-004-0595-3 15711964

[B58] Hashemi H. F. Goodman J. M. (2015). The life cycle of lipid droplets. Curr. Opin. Cell Biol. 33, 119–124. 10.1016/j.ceb.2015.02.002 25703629 PMC4380764

[B59] Hauser F. E. van Hazel I. Chang B. S. (2014). Spectral tuning in vertebrate short wavelength-sensitive 1 (SWS1) visual pigments: can wavelength sensitivity be inferred from sequence data? J. Exp. Zool. B Mol. Dev. Evol. 322, 529–539. 10.1002/jez.b.22576 24890094

[B60] Hemmi J. M. Grünert U. (1999). Distribution of photoreceptor types in the retina of a marsupial, the tammar wallaby (*Macropus eugenii*). Visual Neurosci. 16, 291–302. 10.1017/s0952523899162102 10367964

[B61] Hemmi J. M. Maddess T. Mark R. F. (2000). Spectral sensitivity of photoreceptors in an Australian marsupial, the tammar wallaby (*Macropus eugenii*). Vision Res. 40, 591–599. 10.1016/s0042-6989(99)00210-2 10824263

[B62] Hill G. E. Inouye C. Y. Montgomerie R. (2002). Dietary carotenoids predict plumage coloration in wild house finches. Proc. R. Soc. Lond. B Biol. Sci. 269, 1119–1124. 10.1098/rspb.2002.1980 12061954 PMC1691014

[B63] Hisatomi O. Kayada S. Taniguchi Y. Kobayashi Y. Satoh T. Tokunaga F. (1998). Primary structure and characterization of a bullfrog visual pigment contained in small single cones. Comp. Biochem. Physiol. B Biochem. Mol. Biol. 119, 585–591. 10.1016/s0305-0491(98)00032-7 9734342

[B64] Irisarri I. Baurain D. Brinkmann H. Delsuc F. Sire J. Y. Kupfer A.. (2017). Phylotranscriptomic consolidation of the jawed vertebrate timetree. Nat. Ecol. Evol. 1, 1370–1378. 10.1038/s41559-017-0240-5 28890940 PMC5584656

[B65] Ives J. T. Normann R. A. Barber P. W. (1983). Light intensification by cone oil droplets: electromagnetic considerations. J. Opt. Soc. Am. 73, 1725–1731. 10.1364/josa.73.001725

[B66] Jacobs G. H. (1993). The distribution and nature of colour vision among the mammals. Biol. Rev. Camb. Philos. Soc. 68, 413–471. 10.1111/j.1469-185x.1993.tb00738.x 8347768

[B67] Jacobs G. H. (2009). Evolution of colour vision in mammals. Philos. Trans. R. Soc. Lond. B Biol. Sci. 364, 2957–2967. 10.1098/rstb.2009.0039 19720656 PMC2781854

[B68] Johnston D. Hudson R. A. (1974). Phospholipids of the cone-rich chicken retina and its photoreceptor outer segment membranes. Biochim. Biophys. Acta 369, 269–277. 10.1016/0005-2760(74)90141-6

[B69] Johnston D. Hudson R. A. (1976). Isolation and composition of the carotenoid-containing oil droplets from cone photoreceptors. Biochim. Biophys. Acta 424, 235–245. 10.1016/0005-2760(76)90191-0 1252489

[B70] Knabe W. Skatchkov S. Kuhn H. J. (1997). “Lens mitochondria” in the retinal cones of the tree-shrew *Tupaia belangeri*. Vision Res. 37, 267–271. 10.1016/s0042-6989(96)00199-x 9135860

[B71] Knott B. Berg M. L. Morgan E. R. Buchanan K. L. Bowmaker J. K. Bennett A. T. (2010). Avian retinal oil droplets: dietary manipulation of colour vision? Proc. R. Soc. 277, 953–962. 10.1098/rspb.2009.1805 19939843 PMC2842729

[B72] Koushan K. Rusovici R. Li W. Ferguson L. R. Chalam K. V. (2013). The role of lutein in eye-related disease. Nutrients 5, 1823–1839. 10.3390/nu5051823 23698168 PMC3708350

[B73] Kram Y. A. Mantey S. Corbo J. C. (2010). Avian cone photoreceptors tile the retina as five independent, self-organizing mosaics. PLoS One 5:e8992. 10.1371/journal.pone.0008992 20126550 PMC2813877

[B74] Krinsky N. I. Landrum J. T. Bone R. A. (2003). Biologic mechanisms of the protective role of lutein and zeaxanthin in the eye. Annu. Rev. Nutr. 23, 171–201. 10.1146/annurev.nutr.23.011702.073307 12626691

[B75] Liebman P. A. Entine G. (1968). Visual pigments of frog and tadpole (*Rana pipiens*). Vision Res. 8, 761–775. 10.1016/0042-6989(68)90128-4 5664012

[B76] Liebman P. A. Granda A. M. (1971). Microspectrophotometric measurements of visual pigments in two species of turtle, *Pseudemys scripta* and *Chelonia mydas*. Vision Res. 11, 105–114. 10.1016/0042-6989(71)90227-6 5551491

[B77] Liebman P. A. Granda A. M. (1975). Super dense carotenoid spectra resolved in single cone oil droplets. Nature 253, 370–372. 10.1038/253370a0 1110782

[B78] Lim H. H. Pike T. W. (2016). Dietary carotenoid availability affects avian color discrimination. Behav. Ecol. 27, 1579–1584. 10.1093/beheco/arw116

[B80] Lind O. Chavez J. Kelber A. (2014). The contribution of single and double cones to spectral sensitivity in budgerigars during changing light conditions. J. Comp. Physiol. A Neuroethol. Sens. Neural Behav. Physiol. 200, 197–207. 10.1007/s00359-013-0878-7 24366429

[B79] Lind O. Kelber A. (2009). The intensity threshold of colour vision in two species of parrot. J. Exp. Biol. 212, 3693–3699. 10.1242/jeb.035477 19880731

[B81] Lind O. Kelber A. (2011). The spatial tuning of achromatic and chromatic vision in budgerigars. J. Vis. 11:2. 10.1167/11.7.2 21636524

[B82] Lluch S. Lãpez-Fuster M. J. Ventura J. (2003). Giant mitochondria in the retina cone inner segments of shrews of genus *Sorex* (Insectivora, Soricidae). Anat. Rec. A Discov. Mol. Cell. Evol. Biol. 272, 484–490. 10.1002/ar.a.10066 12740941

[B84] Loew E. R. Fleishman L. J. Foster R. G. Provencio I. (2002). Visual pigments and oil droplets in diurnal lizards: a comparative study of Caribbean anoles. J. Exp. Biol. 205, 927–938. 11916989 10.1242/jeb.205.7.927

[B83] Loew E. R. Govardovskii V. I. (2001). Photoreceptors and visual pigments in the red-eared turtle, *Trachemys scripta elegans*. Vis. Neurosci. 18, 753–757. 10.1017/s0952523801185081 11925010

[B85] Lopes R. J. Johnson J. D. Toomey M. B. Ferreira M. S. Araujo P. M. Melo-Ferreira J.. (2016). Genetic basis for red coloration in birds. Curr. Biol. 26, 1427–1434. 10.1016/j.cub.2016.03.076 27212400 PMC5125026

[B86] López R. López-Gallardo M. Busturia I. Anezary L. Prada C. (2005). Spatial and temporal patterns of growth and differentiation of cone oil droplets in the chick retina. J. Neurosci. Res. 79, 401–411. 10.1002/jnr.20360 15605374

[B87] Lyons B. Karuso P. Jamie J. F. Simpanya M. F. Giblin F. Truscott R. J. W. (2014). Characterisation of a novel UV filter in the lens of the thirteen-lined ground squirrel (*Ictidomys tridecemlineatus*). Exp. Eye Res. 121, 114–120. 10.1016/j.exer.2014.01.022 24530498

[B88] MacNichol E. F. Jr. Kunz Y. W. Levine J. S. Harosi F. I. Collins B. A. (1978). Ellipsosomes: organelles containing a cytochrome-like pigment in the retinal cones of certain fishes. Science 200, 549–552. 10.1126/science.644317 644317

[B89] Mandelman T. Sivak J. G. (1983). Longitudinal chromatic aberration of the vertebrate eye. Vision Res. 23, 1555–1559. 10.1016/0042-6989(83)90169-4 6666057

[B90] Mariani A. P. Leure-duPree A. E. (1978). Photoreceptors and oil droplet colors in the red area of the pigeon retina. J. Comp. Neurol. 182, 821–837. 10.1002/cne.901820506 730850

[B91] Masuda T. Wada Y. Kawamura S. (2016). ES1 is a mitochondrial enlarging factor contributing to form mega-mitochondria in zebrafish cones. Sci. Rep. 6:22360. 10.1038/srep22360 26926452 PMC4772128

[B92] McGraw K. J. Ardia D. R. (2003). Carotenoids, immunocompetence, and the information content of sexual colors: an experimental test. Am. Nat. 162, 704–712. 10.1086/378904 14737708

[B93] Meyer D. B. Stuckey S. R. Hudson R. A. (1971). Oil droplet carotenoids of avian cones. I. Dietary exclusion: models for biochemical and physiological studies. Comp. Biochem. Physiol. B 40, 61–70. 10.1016/0305-0491(71)90062-9 4334820

[B94] Meyer-Rochow V. B. Wohlfahrt S. Ahnelt P. K. (2005). Photoreceptor cell types in the retina of the tuatara (*Sphenodon punctatus*) have cone characteristics. Micron 36, 423–428. 10.1016/j.micron.2005.03.009 15896966

[B95] Morris V. B. Shorey C. D. (1967). An electron microscope study of types of receptor in the chick retina. J. Comp. Neurol. 129, 313–340. 10.1002/cne.901290404 6033986

[B96] Mundy N. I. Stapley J. Bennison C. Tucker R. Twyman H. Kim K. W.. (2016). Red carotenoid coloration in the zebra finch is controlled by a cytochrome P450 gene cluster. Curr. Biol. 26, 1435–1440. 10.1016/j.cub.2016.04.047 27212402

[B97] Muntz W. (1972). “Inert absorbing and reflecting pigments,” in Handbook of Sensory Physiology, (Vol. 7) ed. Dartnall H. J. A. (Berlin: Springer-Verlag), 529–565.

[B98] Nag T. C. Bhattacharjee J. (1995). Retinal ellipsosomes: morphology, development, identification, and comparison with oil droplets. Cell Tissue Res. 279, 633–637. 10.1007/bf00318176 7736559

[B99] Neumeyer C. Jäger J. (1985). Spectral sensitivity of the freshwater turtle *Pseudemys scripta elegans*: evidence for the filter-effect of colored oil droplets. Vision Res. 25, 833–838. 10.1016/0042-6989(85)90191-9 4024481

[B100] O’Day K. (1936). A preliminary note on the presence of double cones and oil droplets in the retina of marsupials. J. Anat. 70, 465–467. 17104608 PMC1249140

[B101] Odeen A. Hastad O. (2003). Complex distribution of avian color vision systems revealed by sequencing the SWS1 opsin from total DNA. Mol. Biol. Evol. 20, 855–861. 10.1093/molbev/msg108 12716987

[B102] Ödeen A. Håstad O. (2013). The phylogenetic distribution of ultraviolet sensitivity in birds. BMC Evol. Biol. 13:36. 10.1186/1471-2148-13-36 23394614 PMC3637589

[B103] Ohtsuka T. (1984). Fluorescence from colorless oil droplets: a new criterion for identification of cone photoreceptors. Neurosci. Lett. 52, 241–245. 10.1016/0304-3940(84)90168-x 6521968

[B104] Olsson P. Lind O. Kelber A. (2015). Bird colour vision: behavioural thresholds reveal receptor noise. J. Exp. Biol. 218, 184–193. 10.1242/jeb.111187 25609782

[B105] Olsson P. Wilby D. Kelber A. (2016). Quantitative studies of animal colour constancy: using the chicken as model. Proc. Biol. Sci. 283:20160411. 10.1098/rspb.2016.0411 27170714 PMC4874714

[B106] Onal G. Kutlu O. Gozuacik D. Dokmeci Emre S. (2017). Lipid droplets in health and disease. Lipids Health Dis. 16:128. 10.1186/s12944-017-0521-7 28662670 PMC5492776

[B107] Pedler C. Boyle M. (1969). Multiple oil droplets in the photoreceptors of the pigeon. Vision Res. 9, 525–528. 10.1016/0042-6989(69)90140-0 5822019

[B108] Peiponen V. A. (1964). Zur Bedeutung der Ölkugeln im Farbensehen der Sauropsiden. Ann. Zool. Fenn. 1, 281–302.

[B109] Provencio I. Loew E. R. Foster R. G. (1992). Vitamin A_2_-based visual pigments in fully terrestrial vertebrates. Vision Res. 32, 2201–2208. 10.1016/0042-6989(92)90084-v 1287997

[B110] Roaf H. (1929). The absorption of light by the coloured globules in the retina of the domestic hen. Proc. R. Soc. Lond. B Biol. Sci. 105, 371–374. 10.1098/rspb.1929.0049

[B111] Roaf H. E. (1933). Colour vision. Physiol. Rev. 13, 43–79.

[B112] Robinson S. R. (1994). Early vertebrate color-vision. Nature 367, 121–121. 10.1038/367121a08114909

[B113] Rohlich P. Szel A. (2000). Photoreceptor cells in the Xenopus retina. Microsc. Res. Tech. 50, 327–337. 10.1002/1097-0029(20000901)50:5<327::aid-jemt2>3.3.co;2-g 10941169

[B114] Röll B. (2000). Characterization of retinal oil droplets in diurnal geckos (Reptilia, Gekkonidae). J. Exp. Zool. 287, 467–476. 10.1002/1097-010x(20001201)287:7<467::aid-jez2>3.0.co;2-8 11110160

[B115] Röll B. (2001). Gecko vision—retinal organization, foveae and implications for binocular vision. Vision Res. 41, 2043–2056. 10.1016/s0042-6989(01)00093-1 11403789

[B116] Schiedt K. (1998). “Absorption and metabolism of carotenoids in birds, fish, and crustaceans,” in Carotenoids Volume 3: Biosynthesis and Metabolism, eds Britton G. Liaaen-Jensen S. Pfander H. (Basel, Switzerland: Birkhäuser Verlag), 285–358.

[B117] Schiedt K. Bischof S. Glinz E. (1991). Recent progress on carotenoid metabolism in animals. Pure Appl. Chem. 63, 89–100. 10.1351/pac199163010089

[B118] Seddon J. M. Ajani U. A. Sperduto R. D. Hiller R. Blair N. Burton T. C.. (1994). Dietary carotenoids, vitamins A, C, and E, and advanced age-related macular degeneration. Eye Disease Case-Control Study Group. JAMA 272, 1413–1420. 10.1001/jama.272.18.1413 7933422

[B119] Shi Y. Radlwimmer F. B. Yokoyama S. (2001). Molecular genetics and the evolution of ultraviolet vision in vertebrates. Proc. Natl. Acad. Sci. U S A 98, 11731–11736. 10.1073/pnas.201257398 11573008 PMC58798

[B120] Siddiqi A. Cronin T. W. Loew E. R. Vorobyev M. Summers K. (2004). Interspecific and intraspecific views of color signals in the strawberry poison frog *Dendrobates pumilio*. J. Exp. Biol. 207, 2471–2485. 10.1242/jeb.01047 15184519

[B122] Sillman A. J. Beach A. K. Dahlin D. A. Loew E. R. (2005). Photoreceptors and visual pigments in the retina of the fully anadromous green sturgeon (*Acipenser medirostrus*) and the potamodromous pallid sturgeon (*Scaphirhynchus albus*). J. Comp. Physiol. A Neuroethol. Sens. Neural Behav. Physiol. 191, 799–811. 10.1007/s00359-005-0004-6 15983809

[B121] Sillman A. J. Dahlin D. A. (2004). “The photoreceptors and visual pigments of sharks and sturgeons,” in The Senses of Fish, eds Von Der Emde G. Mogdans J. Kapoor B. G. (Dordrecht: Springer Netherlands), 31–54.

[B123] Sillman A. J. Govardovskii V. I. Röhlich P. Southard J. A. Loew E. R. (1997). The photoreceptors and visual pigments of the garter snake (*Thamnophis sirtalis*): a microspectrophotometric, scanning electron microscopic and immunocytochemical study. J. Comp. Physiol. A 181, 89–101. 10.1007/s003590050096 9251253

[B124] Sillman A. J. O’Leary C. J. Tarantino C. D. Loew E. R. (1999). The photoreceptors and visual pigments of two species of Acipenseriformes, the shovelnose sturgeon (*Scaphirhynchus platorynchus*) and the paddlefish (*Polyodon spathula*). J. Comp. Physiol. A Neuroethol. Sens. Neural Behav. Physiol. 184, 37–47. 10.1007/s003590050304

[B125] Sillman A. J. Ronan S. J. Loew E. R. (1991). Histology and microspectrophotometry of the photoreceptors of a crocodilian, *Alligator mississippiensis*. Proc. R. Soc. Lond. B Biol. Sci. 243, 93–98. 10.1098/rspb.1991.0016

[B126] Sillman A. J. Sorsky M. E. Loew E. R. (1995). The visual pigments of wild white sturgeon (*Acipenser transmontanus*). Can. J. Zool. 73, 805–809. 10.1139/z95-093

[B127] Sillman A. J. Spanfelner M. D. Loew E. R. (1990). The photoreceptors and visual pigments in the retina of the white sturgeon, *Acipenser transmontanus*. Can. J. Zool. 68, 1544–1551. 10.1139/z90-228

[B128] Smith R. L. Nishimura Y. Raviola G. (1985). Interreceptor junction in the double cone of the chicken retina. J. Submicrosc. Cytol. 17, 183–186. 3999181

[B129] Snodderly D. M. Auran J. D. Delori F. C. (1984a). The macular pigment. II. Spatial distribution in primate retinas. Invest. Ophthalmol. Vis. Sci. 25, 674–685. 6724837

[B130] Snodderly D. M. Brown P. K. Delori F. C. Auran J. D. (1984b). The macular pigment. I. Absorbance spectra, localization, and discrimination from other yellow pigments in primate retinas. Invest. Ophthalmol. Vis. Sci. 25, 660–673. 6724836

[B131] Stavenga D. G. Wilts B. D. (2014). Oil droplets of bird eyes: microlenses acting as spectral filters. Philos. Trans. R. Soc. Lond. B Biol. Sci. 369:20130041. 10.1098/rstb.2013.0041 24395968 PMC3886329

[B132] Stoddard M. C. Prum R. O. (2008). Evolution of avian plumage color in a tetrahedral color space: a phylogenetic analysis of new world buntings. Am. Nat. 171, 755–776. 10.1086/587526 18419340

[B133] Strother G. K. (1963). Absorption spectra of retinal oil globules in turkey, turtle and pigeon. Exp. Cell Res. 29, 349–355. 10.1016/0014-4827(63)90389-6 13978950

[B134] Sun H. J. Frost B. J. (1997). Motion processing in pigeon tectum: equiluminant chromatic mechanisms. Exp. Brain Res. 116, 434–444. 10.1007/pl00005771 9372292

[B135] Svensson P. A. Wong B. B. M. (2011). Carotenoid-based signals in behavioural ecology: a review. Behaviour 148, 131–189. 10.1163/000579510x548673

[B136] Tarboush R. Novales Flamarique I. Chapman G. B. Connaughton V. P. (2014). Variability in mitochondria of zebrafish photoreceptor ellipsoids. Vis. Neurosci. 31, 11–23. 10.1017/s095252381300059x 24801620

[B137] Thomson L. R. Toyoda Y. Delori F. C. Garnett K. M. Wong Z. Y. Nichols C. R.. (2002a). Long term dietary supplementation with zeaxanthin reduces photoreceptor death in light-damaged Japanese quail. Exp. Eye Res. 75, 529–542. 10.1006/exer.2002.2050 12457865

[B138] Thomson L. R. Toyoda Y. Langner A. Delori F. C. Garnett K. M. Craft N.. (2002b). Elevated retinal zeaxanthin and prevention of light-induced photoreceptor cell death in quail. Invest. Ophthalmol. Vis. Sci. 43, 3538–3549. 12407166

[B139] Toews D. P. Hofmeister N. R. Taylor S. A. (2017). The evolution and genetics of carotenoid processing in animals. Trends Genet. 33, 171–182. 10.1016/j.tig.2017.01.002 28174022

[B144] Toomey M. B. Butler M. W. Mcgraw K. J. (2010). Immune-system activation depletes retinal carotenoids in house finches (*Carpodacus mexicanus*). J. Exp. Biol. 213, 1709–1716. 10.1242/jeb.041004 20435822

[B145] Toomey M. B. Collins A. M. Frederiksen R. Cornwall M. C. Timlin J. A. Corbo J. C. (2015). A complex carotenoid palette tunes avian colour vision. J. R. Soc. Interface 12:20150563. 10.1098/rsif.2015.0563 26446559 PMC4614492

[B146] Toomey M. B. Lind O. Frederiksen R. Curley R. W. Jr. Riedl K. M. Wilby D.. (2016). Complementary shifts in photoreceptor spectral tuning unlock the full adaptive potential of ultraviolet vision in birds. Elife 5:e15675. 10.7554/elife.15675 27402384 PMC4947394

[B147] Toomey M. B. Lopes R. J. Araújo P. M. Johnson J. D. Gazda M. A. Afonso S.. (2017). High-density lipoprotein receptor SCARB1 is required for carotenoid coloration in birds. Proc. Natl. Acad. Sci. U S A 114, 5219–5224. 10.1073/pnas.1700751114 28465440 PMC5441819

[B140] Toomey M. B. McGraw K. J. (2010). The effects of dietary carotenoid intake on carotenoid accumulation in the retina of a wild bird, the house finch (*Carpodacus mexicanus*). Arch. Biochem. Biophys. 504, 161–168. 10.1016/j.abb.2010.06.033 20599667

[B141] Toomey M. B. McGraw K. J. (2011). The effects of dietary carotenoid supplementation and retinal carotenoid accumulation on vision-mediated foraging in the house finch. PLoS One 6:e21653. 10.1371/journal.pone.0021653 21747917 PMC3126843

[B142] Toomey M. B. McGraw K. J. (2012). Mate choice for a male carotenoid-based ornament is linked to female dietary carotenoid intake and accumulation. BMC Evol. Biol. 12:3. 10.1186/1471-2148-12-3 22233462 PMC3315416

[B143] Toomey M. B. McGraw K. J. (2016). The effects of sun exposure on carotenoid accumulation and oxidative stress in the retina of the House finch (*Haemorhous mexicanus*). Avian Res. 7:5 10.1186/s40657-016-0041-6

[B148] Twyman H. Valenzuela N. Literman R. Andersson S. Mundy N. I. (2016). Seeing red to being red: conserved genetic mechanism for red cone oil droplets and co-option for red coloration in birds and turtles. Proc. R. Soc. Lond. B Biol. Sci. 283:20161208. 10.1098/rspb.2016.1208 27488652 PMC5013772

[B149] Vlahos L. M. Knott B. Valter K. Hemmi J. M. (2014). Photoreceptor topography and spectral sensitivity in the common brushtail possum (*Trichosurus vulpecula*). J. Comp. Neurol. 522, 3423–3436. 10.1002/cne.23610 24737644

[B150] Vorobyev M. (2003). Coloured oil droplets enhance colour discrimination. Proc. R. Soc. Lond. B Biol. Sci. 270, 1255–1261. 10.1098/rspb.2003.2381 12816638 PMC1691374

[B151] Vorobyev M. Osorio D. Bennett A. T. Marshall N. J. Cuthill I. C. (1998). Tetrachromacy, oil droplets and bird plumage colours. J. Comp. Physiol. A 183, 621–633. 10.1007/s003590050286 9839454

[B152] Waelchli G. (1883). Zur Topographie der gefärbten Kugeln der Vogelnetzhaut. Albrecht Von Graefes Arch. Ophthalmol. 29, 205–223. 10.1007/bf01692872

[B153] Wald G. (1938). On rhodopsin in solution. J. Gen. Physiol. 21, 795–832. 10.1085/jgp.21.6.795 19873085 PMC2141975

[B154] Wald G. (1948). Galloxanthin, a carotenoid from the chicken retina. J. Gen. Physiol. 31, 377–383. 10.1085/jgp.31.5.377 18917022 PMC2147121

[B155] Wald G. Zussman H. (1938). Carotenoids of the chicken retina. J. Biol. Chem. 122, 449–460.

[B156] Walls G. L. (1934). The reptilian retina. Am. J. Ophthalmol. 17, 892–915. 10.1016/s0002-9394(34)93309-2

[B157] Walls G. L. (1939). Notes on the retinae of two opossum genera. J. Morphol. 64, 67–87. 10.1002/jmor.1050640106

[B158] Walls G. L. (1942). The Vertebrate Eye and its Adaptive Radiation. Bloomfield Hills, MI: Cranbrook Institute of Science.

[B159] Walls G. L. Judd H. D. (1933). The intra-ocular colour-filters of vertebrates. Br. J. Ophthalmol. 17, 641–675. 10.1136/bjo.17.11.641 18169162 PMC511614

[B160] Welte M. A. (2015). Expanding roles for lipid droplets. Curr. Biol. 25, R470–R481. 10.1016/j.cub.2015.04.004 26035793 PMC4452895

[B161] Wilby D. Roberts N. W. (2017). Optical influence of oil droplets on cone photoreceptor sensitivity. J. Exp. Biol. 220, 1997–2004. 10.1242/jeb.152918 28314749 PMC5482973

[B162] Wilby D. Toomey M. B. Olsson P. Frederiksen R. Cornwall M. C. Oulton R.. (2015). Optics of cone photoreceptors in the chicken (*Gallus gallus domesticus*). J. R. Soc. Interface 12:20150591. 10.1098/rsif.2015.0591 26423439 PMC4614498

[B163] Wong R. O. (1989). Morphology and distribution of neurons in the retina of the American garter snake *Thamnophis sirtalis*. J. Comp. Neurol. 283, 587–601. 10.1002/cne.902830412 2745756

[B164] Worthey J. A. Brill M. H. (1986). Heuristic analysis of von Kries color constancy. J. Opt. Soc. Am. A 3, 1708–1712. 10.1364/josaa.3.001708 3772633

[B165] Yokoyama S. Zhang H. Radlwimmer F. B. Blow N. S. (1999). Adaptive evolution of color vision of the Comoran coelacanth (*Latimeria chalumnae*). Proc. Natl. Acad. Sci. U S A 96, 6279–6284. 10.1073/pnas.96.11.6279 10339578 PMC26872

[B167] Young S. R. Martin G. R. (1984). Optics of retinal oil droplets: a model of light collection and polarization detection in the avian retina. Vision Res. 24, 129–137. 10.1016/0042-6989(84)90098-1 6710875

[B166] Young H. M. Pettigrew J. D. (1991). Cone photoreceptors lacking oil droplets in the retina of the echidna, *Tachyglossus aculeatus* (Monotremata). Vis. Neurosci. 6, 409–420. 10.1017/s0952523800001279 2069895

[B168] Zeiss C. J. Schwab I. R. Murphy C. J. Dubielzig R. W. (2011). Comparative retinal morphology of the platypus. J. Morphol. 272, 949–957. 10.1002/jmor.10959 21567446

[B169] Zigman S. Paxhia T. (1988). The nature and properties of squirrel lens yellow pigment. Exp. Eye Res. 47, 819–824. 10.1016/0014-4835(88)90065-6 3215294

